# Symmetry and simplicity spontaneously emerge from the algorithmic nature of evolution

**DOI:** 10.1073/pnas.2113883119

**Published:** 2022-03-11

**Authors:** Iain G. Johnston, Kamaludin Dingle, Sam F. Greenbury, Chico Q. Camargo, Jonathan P. K. Doye, Sebastian E. Ahnert, Ard A. Louis

**Affiliations:** ^a^Department of Mathematics, University of Bergen, Bergen 5007, Norway;; ^b^Computational Biology Unit, University of Bergen, Bergen 5008, Norway;; ^c^Rudolf Peierls Centre for Theoretical Physics, University of Oxford, Oxford OX1 3PU, United Kingdom;; ^d^The Alan Turing Institute, British Library, London NW1 2DB, United Kingdom;; ^e^Centre for Applied Mathematics and Bioinformatics, Department of Mathematics and Natural Sciences, Gulf University for Science and Technology, Hallwaly, Kuwait;; ^f^Theory of Condensed Matter Group, Cavendish Laboratory, University of Cambridge, Cambridge CB3 0HE, United Kingdom;; ^g^Department of Metabolism, Digestion and Reproduction, Imperial College London, London SW7 2AZ, United Kingdom;; ^h^Department of Computer Science, University of Exeter, Exeter EX4 4QF, United Kingdom;; ^i^Physical and Theoretical Chemistry Laboratory, Department of Chemistry, University of Oxford, Oxford OX1 3QZ, United Kingdom;; ^j^Department of Chemical Engineering and Biotechnology, University of Cambridge, Cambridge CB3 0AS, United Kingdom

**Keywords:** evolution, development, algorithmic information theory

## Abstract

Engineers routinely design systems to be modular and symmetric in order to increase robustness to perturbations and to facilitate alterations at a later date. Biological structures also frequently exhibit modularity and symmetry, but the origin of such trends is much less well understood. It can be tempting to assume—by analogy to engineering design—that symmetry and modularity arise from natural selection. However, evolution, unlike engineers, cannot plan ahead, and so these traits must also afford some immediate selective advantage which is hard to reconcile with the breadth of systems where symmetry is observed. Here we introduce an alternative nonadaptive hypothesis based on an algorithmic picture of evolution. It suggests that symmetric structures preferentially arise not just due to natural selection but also because they require less specific information to encode and are therefore much more likely to appear as phenotypic variation through random mutations. Arguments from algorithmic information theory can formalize this intuition, leading to the prediction that many genotype–phenotype maps are exponentially biased toward phenotypes with low descriptional complexity. A preference for symmetry is a special case of this bias toward compressible descriptions. We test these predictions with extensive biological data, showing that protein complexes, RNA secondary structures, and a model gene regulatory network all exhibit the expected exponential bias toward simpler (and more symmetric) phenotypes. Lower descriptional complexity also correlates with higher mutational robustness, which may aid the evolution of complex modular assemblies of multiple components.

## Significance

Why does evolution favor symmetric structures when they only represent a minute subset of all possible forms? Just as monkeys randomly typing into a computer language will preferentially produce outputs that can be generated by shorter algorithms, so the coding theorem from algorithmic information theory predicts that random mutations, when decoded by the process of development, preferentially produce phenotypes with shorter algorithmic descriptions. Since symmetric structures need less information to encode, they are much more likely to appear as potential variation. Combined with an arrival-of-the-frequent mechanism, this algorithmic bias predicts a much higher prevalence of low-complexity (high-symmetry) phenotypes than follows from natural selection alone and also explains patterns observed in protein complexes, RNA secondary structures, and a gene regulatory network.

Evolution proceeds through genetic mutations which generate the novel phenotypic variation upon which natural selection can act. The relationship between the space of genotypes and the space of phenotypes can be encapsulated as a genotype–phenotype (GP) map (1–3). These can be viewed algorithmically, where random genetic mutations search in the space of (developmental) algorithms encoded by the GP map, a relationship that has been highlighted, for example, in plants (4), in Dawkins’ “biomorphs” (5), and in biomolecules (6).

Genetic mutations are random in the sense that they occur independently of the phenotypic variation they produce. This does not, however, mean that the probability *P*(*p*) that a GP map produces a phenotype *p* upon random sampling of genotypes will be anything like a uniformly random distribution. Instead, highly general (but rather abstract) arguments based on the coding theorem of algorithmic information theory (AIT) (7) predict that the *P*(*p*) of many GP maps should be highly biased toward phenotypes with low Kolmogorov complexity *K*(*p*) (8). High symmetry can, in turn, be linked to low *K*(*p*) (6, 9–11). An intuitive explanation for this algorithmic bias toward symmetry proceeds in two steps: 1) Symmetric phenotypes typically need less information to encode algorithmically, due to repetition of subunits. This higher compressibility reduces constraints on genotypes, implying that more genotypes will map to simpler, more symmetric phenotypes than to more complex asymmetric ones (2, 3). 2) Upon random mutations these symmetric phenotypes are much more likely to arise as potential variation (12, 13), so that a strong bias toward symmetry may emerge even without natural selection for symmetry.

## Symmetry in Protein Quaternary Structure and Polyominoes

**Fig. 1.**
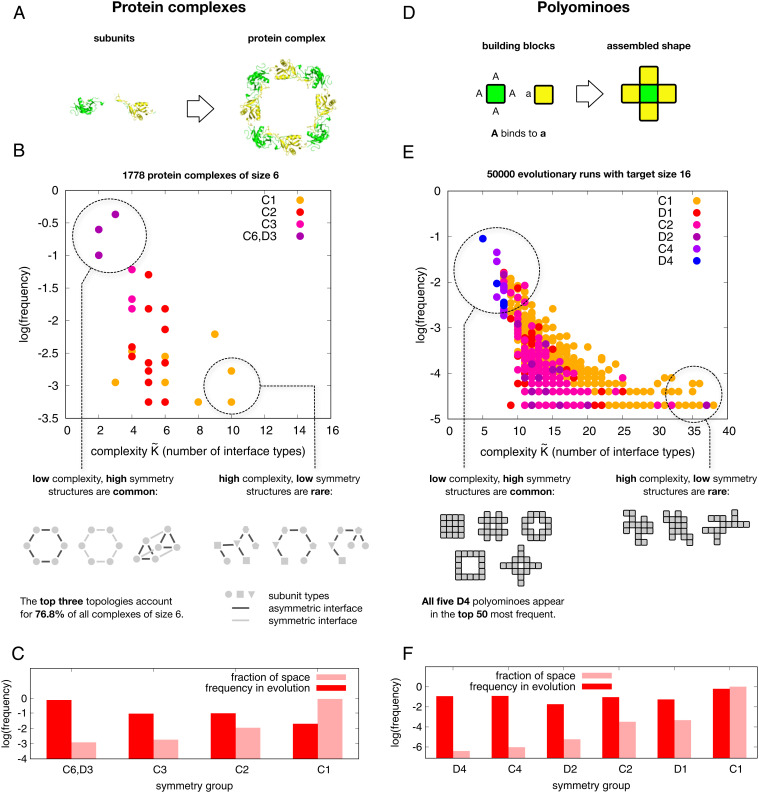
(*A*) Protein complexes self-assemble from individual units. (*B*) Frequency of 6-mer protein complex topologies found in the PDB versus the number of interface types, a measure of complexity $\tilde{K}(p)$. Symmetry groups are in standard Schoenflies notation: *C*_6_, *D*_3_, *C*_3_, *C*_2_, and *C*_1_. There is a strong preference for low-complexity/high-symmetry structures. (*C*) Histograms of scaled frequencies of symmetries for 6-mer topologies found in the PDB (dark red) versus the frequencies by symmetry of the morphospace of all possible 6-mers illustrate that symmetric structures are hugely overrepresented in the PDB database. (*D*) Polyomino complexes self-assemble from individual units (here *a* binds to *A*) just as the proteins do. (*E*) Scaled frequency of polyominoes that fix in evolutionary simulations with a fitness maximum at 16-mers, versus the number of interface types (a measure of complexity $\tilde{K}(p)$) exhibits a strong bias toward high-symmetry structures, similar to protein complexes. (*F*) Histograms of the frequency of symmetry groups for all 16-mers (light) and for 16-mers appearing in the evolutionary runs (dark) quantify how strongly biased variation drives a pronounced preference for high-symmetry structures.

We first explore evidence for this algorithmic hypothesis by studying protein quaternary structure, which describes the multimeric complexes into which many proteins self-assemble in order to perform key cellular functions (Fig. 1*A* and *SI Appendix*, Fig. S1 and section S1). These complexes can form in the cell if proteins evolve attractive interfaces allowing them to bind to each other (14–16). We analyzed a curated set of 34,287 protein complexes extracted from the Protein Data Bank (PDB) that were categorized into 120 different bonding topologies (16). In Fig. 1*B*, we plot, for all complexes involving six subunits (6-mers), the frequency with which a protein complex of topology *p* appears against the descriptional complexity $\tilde{K}(p)$, an approximate measure of its true Kolmogorov assembly complexity *K*(*p*), defined here as the minimal number of distinct interfaces required to assemble the given structure under general self-assembly rules (*Materials and Methods*). This definition of $\tilde{K}(p)$ can also be thought of as a measure of the minimal number of evolutionary innovations needed to make a self-assembling complex. The highest-probability structures all have relatively low $\tilde{K}(p)$. Since structures with higher symmetry need less information to describe (6, 9–11), the most frequently observed complexes are also highly symmetric. Fig. 1*C* and *SI Appendix*, Figs. S2*A* and S3*A* further demonstrate that structures found in the PDB are significantly more symmetric than the set of all possible 6-mers (*Materials and Methods*). Similar biases toward high-symmetry structures obtain for other sizes (*SI Appendix*, Fig. S2*B*).

In order to understand the evolutionary origins of this bias toward symmetry we turn to a tractable GP map for protein quaternary structure. In the Polyomino GP map, two-dimensional tiles self-assemble into polyomino structures (17) that model protein complex topologies (18) (Fig. 1*D*). The sides represent the interfaces that bind proteins together. Within the Polyomino GP map, the genomes are bit strings used to describe a set of tiles and their interactions. The phenotypes are polyomino shapes *p* that emerge from the self-assembly process. Although this model is highly simplified, it has successfully explained evolutionary trends in protein quaternary structure such as the preference of dihedral over cyclic symmetry in homomeric tetramers (15, 17) or the propensity of proteins to form larger aggregates such as hemoglobin aggregation in sickle cell anemia (18).

To explore the strong preference for simple structures, we performed evolutionary simulations where fitness is maximized for polyominoes made of 16 blocks (*Materials and Methods*). With 16 tile types and 64 interface types, the GP map denoted as $S_{16,64}$ allows all 13,079,255 possible 16-mer polyomino topologies (*SI Appendix*, Table 1) to be made. Fig. 1*E* demonstrates that evolutionary outcomes are exponentially biased toward 16-mer structures with low $\tilde{K}(p)$ (using the same complexity measure as for the proteins [*Materials and Methods*]), even though every 16-mer has the same fitness.

The extraordinary strength of the bias toward high symmetry can be further illustrated by examining the prevalence of the two highest-symmetry groups in the outcomes of evolutionary simulations. For 16-mers, there are 5 possible structures in class *D*_4_ (all symmetries of the square) and 12 in *C*_4_ (fourfold rotational symmetry). Even though these 17 structures represent just over a millionth of all 16-mer phenotypes, they make up about 30% of the structures that fix in the evolutionary runs, demonstrating an extremely strong preference for high symmetry (see also *SI Appendix*, Fig. S3*B*). Comparing the histograms in Fig. 1 *C* and *F* shows that the polyominoes exhibit a qualitatively similar bias toward high symmetry as seen for the proteins. We checked that this strong bias toward high symmetry/low $\tilde{K}(p)$ holds for a range of other evolutionary parameters (such as mutation rate) and for other polyomino sizes (*SI Appendix*, Fig. S6 and section S3*C*). Natural selection explains why 16-mers are selected for (as opposed to other sizes). However, since every 16-mer is equally fit, natural selection does not explain the remarkable preference for symmetry observed here, which is instead caused by bias in the arrival of variation.

## Evolutionary Simulations Compared to Sampling

**Fig. 2.**
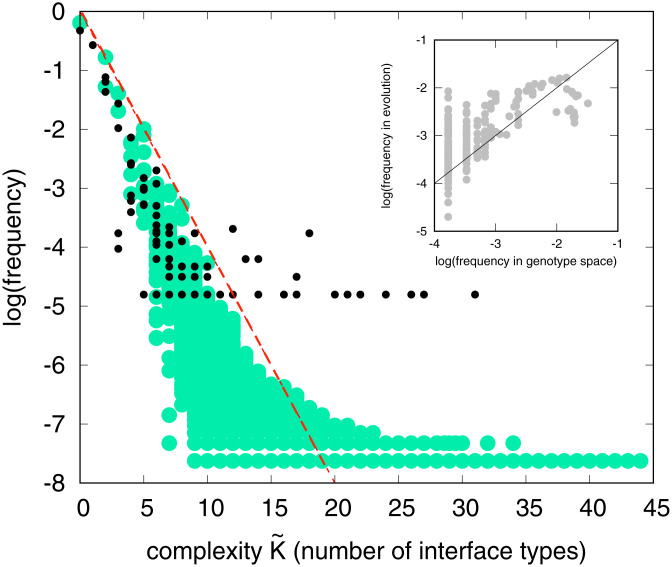
Frequency with which a particular protein quaternary structure topology *p* (black circles) appears in the PDB versus complexity $\tilde{K}(p)$ = number of interface types closely resembles the frequency/*P*(*p*) vs. $\tilde{K}(p)$ distribution of all possible polyomino structures, obtained by randomly sampling 10^8^ genotypes for the $S_{16,64}$ space (green circles). Simpler (more compressible) phenotypes are much more likely to occur. An illustrative AIT upper bound from Eq. **1** is shown with a=0.75,b=0 (dashed red line). (*Inset*) The frequency with which particular 16-mers are found to fix in evolutionary runs from Fig. 1*E* is predicted by the probability *P*(*p*) (or equivalently the frequency) with which they arise on random sampling of genotypes; the solid line denotes *x* = *y*.

In order to further understand the mechanisms that are responsible for the evolutionary preference for high symmetry, we calculated the probability *P*(*p*) of obtaining phenotype (polyomino shape) *p* by uniformly sampling 10^8^ genomes for the $S_{16,64}$ GP map and counting each time a particular structure *p* (which can be any size) appears. Fig. 2 shows that *P*(*p*) (or equivalently the frequency) varies over many orders of magnitude for different *p*. High *P*(*p*) only occurs for low $\tilde{K}(p)$ structures, while high $\tilde{K}(p)$ structures have low *P*(*p*). Fig. 2, *Inset*, shows that the frequency from an evolutionary run from Fig. 1 closely follows the *P*(*p*) for 16-mers from random sampling. We tested this correlation for a range of different evolutionary parameters, and also for both randomly assigned and fixed fitness functions, and always observe relationships between frequency and $\tilde{K}(p)$ that are strikingly similar to those found for random sampling (*SI Appendix*, Fig. S6).

The observed similarity in all these different evolutionary regimes is predicted by the arrival of the frequent population dynamics framework of ref. 12 (*SI Appendix*, section S2). For highly biased GP maps, it predicts that for a wide range of mutation rates and population sizes, the rate at which variation (phenotype *p*) arises in an evolving population is, to first order, directly proportional to the probability *P*(*p*) of it appearing upon uniform random sampling over genotypes. Strong bias in the arrival of variation can overcome fitness differences and so shape evolutionary outcomes (12, 19). Interestingly, recent results for related systems, including the training of deep learning algorithms, support this evolutionary dynamics picture. Deep neural nets show a strong Occam’s razor–like bias toward simple outputs (20) upon random sampling of parameters, and these frequent (and simple) outputs appear with similar probability under training with stochastic gradient descent (21). This similarity between random sampling and the outcome of a stochastic optimizer strengthens the case for extending the applicability of the arrival of the frequent framework for highly biased to maps to a wide range of fitness landscapes (see *SI Appendix*, section S2 for fuller discussion).

Fig. 2 also illustrates a striking similarity between the probability/complexity scaling for polyominoes and that of protein complex structures. Note that finite sampling effects lead to a widening of the lowest-frequency outputs (8) (see also *SI Appendix*, Fig. S5), suggesting that as more structures are deposited in the 3DComplex database (14), the agreement with the polyomino distribution may improve further. Given the simplicity of the polyomino model, the tightness of this quantitative agreement is probably due in part to chance. Nevertheless, the arrival of the frequent mechanism, which for polyominoes explains the remarkably close similarity of the frequency vs. $\tilde{K}(p)$ relationships across different evolutionary scenarios (see, e.g., *SI Appendix*, Fig. S4–S9), predicts that the probability–complexity relationships for the protein complexes will be robust, on average, to the many different evolutionary histories that generated these complexes. Taken together, the data and arguments above strongly favor our hypothesis that bias in the arrival of variation, and not some as yet undiscovered adaptive process, is the first-order explanation of the prevalence of high symmetry in protein complexes.

## AIT and GP Maps

These results beg another question: is the bias toward simplicity (low $\tilde{K}(p)$) observed for protein clusters and polyominoes a more general property of GP maps? Some intuition can be gleaned from the famous trope of monkeys typing at random on typewriters. If each typewriter has *M* keys, then every output of length *N* has equal probability $1/M^{N}$. By contrast, if the monkeys’ keyboard outputs are interpreted as a programming language, then, for example, accidentally hitting the 21 characters of the program “print “01” 500 times;” will generate the N=1,000 -digit string 010101… with probability $1/M^{21}$ instead of $1/M^{1,000}$. In other words, when searching in the space of algorithms, outputs that can be generated by short programs are exponentially more likely to be produced than outputs that can only be generated by long programs.

This intuition that simpler outputs are more likely to appear upon random inputs into a computer programming language can be precisely quantified in the field of AIT (7), where the Kolmogorov complexity *K*(*p*) of a string *p* is formally defined as a shortest program that generates *p* on a suitably chosen universal Turing machine (UTM). While GP maps are typically not UTMs, and strictly speaking, Kolmogorov complexity is uncomputable, a relationship between the probability *P*(*p*) and a computable descriptional complexity $\tilde{K}(p)$ (typically based on compression) which approximates the true *K*(*p*) has recently been derived (8) for (non-UTM) input–output maps f:I→O between *N_I_* inputs and *N_O_* outputs. For a fairly general set of conditions, including that $N_{I}\gg N_{O}$ and that the maps are asymptotically simple (*SI Appendix*, section S5), the probability *P*(*p*) that a map *f* generates output *p* upon random inputs can be bounded as

[1] $$P(p)\leq 2^{-a\tilde{K}(p)-b},$$

where $\tilde{K}(p)$ is an appropriate approximation to the true Kolmogorov complexity *K*(*p*), and *a* and *b* are constants that depend on the map but not on *p*. While Eq. **1** is only an upper bound, it can be shown (22) that outputs generated by uniform random sampling of inputs are likely to be close to the bound. In extensive tests, Eq. **1** provided accurate bounds on the *P*(*p*) for systems ranging from coupled differential equations to the RNA secondary structure (SS) GP map (8) to deep neural networks (20), suggesting widespread applicability.

Since the number of genotypes is typically much greater than the number of phenotypes (1–3), and their relationship is encoded in a set of biophysical rules that typically depend weakly on system size, many GP maps satisfy the conditions (8) for Eq. **1** to apply (see also *SI Appendix*, section S5). In Fig. 2, we show an example of how Eq. **1** can act as an upper bound to *P*(*p*) for the polyominoes and the protein complexes. In *SI Appendix*, section S5*C*, we demonstrate that this AIT formalism also works well for other choices of the complexity $\tilde{K}(p)$, so that our results do not depend on the particular choices we make here. The AIT formalism also suggests that related systems should have similar probability–complexity relationships, which helps explain why the polyominoes and proteins have similar *P*(*p*) vs. $\tilde{K}(p)$ plots.

Since many GP maps satisfy the conditions for simplicity bias, including those where symmetry may be harder to define, we therefore hypothesized that a bias toward simplicity may also strongly affect evolutionary outcomes for many other GP maps. We tested this hypothesis for two other biological examples: RNA secondary structure and a model gene regulatory network (GRN).

## Simplicity Bias in RNA Secondary Structure

Because it can fold into well-defined structures, RNA is a versatile molecule that performs many biologically functional roles besides encoding information. While predicting three-dimensional structure from sequence is hard, a simpler problem of predicting SS, which describes the bonding pattern of the bases, can be both accurately and efficiently calculated (23). The map from sequences to SS is perhaps the best-studied GP map and has provided many conceptual insights into the role of structured variation in evolution (1–3, 12, 24–26). It has already been shown (see, e.g., refs. 25–27) that the highly biased RNA GP map strongly determines the distributions of RNA shape properties in the functional RNA database (fRNAdb) (28) of naturally occurring noncoding RNA (ncRNA). Although natural selection still plays a role (see refs. 25, 26 for further discussions), the dominant determinant of these structural properties is strong bias in the arrival of variation (12). It was recently shown (8) that the RNA SS GP map is well described by Eq. **1**. Combining these observations leads to the hypothesis that functional ncRNA in nature should also be exponentially biased toward more compressible low $\tilde{K}(p)$ structures.

**Fig. 3.**
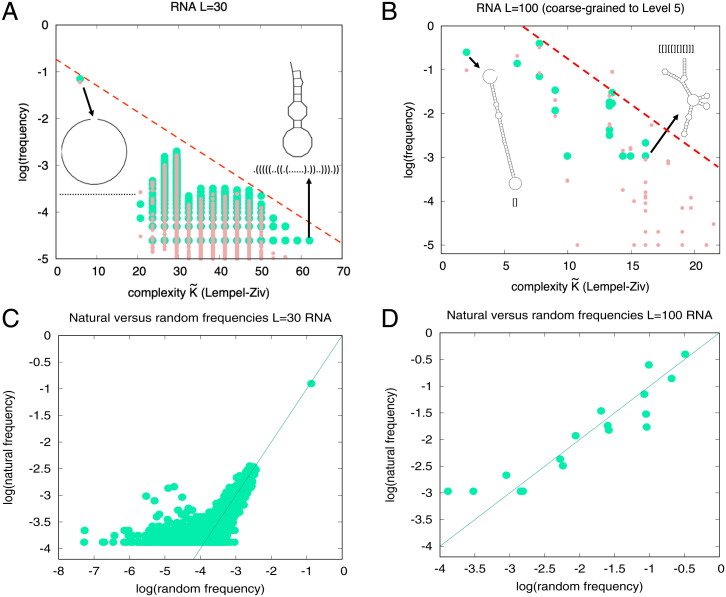
Scaled frequency (occurrence probability) versus complexity $\tilde{K}(p)$ for (*A*) *L* = 30 RNA full SS and (*B*) *L* = 100 SS coarse-grained to level 5 (*Materials and Methods*). Probabilities for structures taken from random sampling of sequences (light red) compare well to the frequency found in the fRNA database (28) (green dots) for 40,554 functional *L* = 30 RNA sequences with 17,603 unique dot-bracket SS and for 932 natural *L* = 100 RNA sequences mapping to 16 unique coarse-grained level 5 structures. The dashed lines show a possible upper bound from Eq. **1**. Examples of high-probability/low-complexity and low-probability/high-complexity SS are also shown. We directly compare the frequency of RNA structures in the fRNAdb database to the frequency of structures upon uniform random sampling of genotypes for (*C*) *L* = 30 SS and (*D*) *L* = 100 coarse-grained structures. The lines are *y* = *x*. Correlation coefficients are 0.71 and 0.92, for *L* = 30 and *L* = 100, respectively, with *P* < $10^{-6}$ for both. Sampling errors are larger at low frequencies.

To test this hypothesis, we first, for RNA sequences of length *L* = 30, calculate $\tilde{K}(p)$ with a standard Lempel–Ziv compression technique (8) to directly measure the descriptional complexity of the dot-bracket notation of an SS (*Materials and Methods* and *SI Appendix*, section S4). Fig. 3*A* shows that there is a strong inverse correlation between frequency and complexity for both naturally occurring and randomly sampled phenotypes [note that *L* = 30 is quite short so that finite size effects are expected (8) to affect the correlation with Eq. **1**]. For longer RNA, the agreement with Eq. **1** is better (see, e.g., ref. 8 and *SI Appendix*, Figs. S10 and S11). For *L* = 30, there are about $3\times 10^{6}$ possible SS (25), but only 17,603 are found in the fRNAdb database (28), and these are much more likely to be more compressible low $\tilde{K}(p)$ structures. Fig. 3*C* shows that randomly sampling sequences provides a good predictor for the frequency with which these structures are found in the database, consistent with previous observations (25, 26) and the arrival of the frequent framework (12).

For lengths longer than *L* = 30, the databases of natural RNAs show little to no repeated SS, so individual frequencies cannot be extracted. To make progress, we apply a well-established coarse-graining strategy that recursively groups together RNA structures by basic properties of their shapes (29), which was applied to naturally occurring RNA SS in ref. 26. At the highest level of coarse-graining (level 5), there are many repeat structures in the fRNAdb database, allowing for frequencies to be directly measured (*Materials and Methods*). For *L* = 100 we compare the empirical frequencies to *P*(*p*) estimated by random sampling. Fig. 3*B* shows that there is again a strong negative correlation between frequency and complexity (see also *SI Appendix*, Tables II and III and Figs. S10 and S11). Fig. 3*D* shows that natural frequencies are well predicted by random sampling, as seen in ref. 26 for other lengths. Again, only a tiny fraction ($\approx 1/10^{8}$) of all possible phenotypes is explored by nature (26). The RNA SS GP map exhibits simplicity bias phenomenology similar to the protein complexes and the polyomino GP map. While the simpler group theory–based symmetries discussed for protein complexes and polyominoes do not apply here, the bias toward lower $\tilde{K}(p)$ reflects the more generalized symmetries in the RNA SS structures.

## Model Gene Regulatory Network

**Fig. 4.**
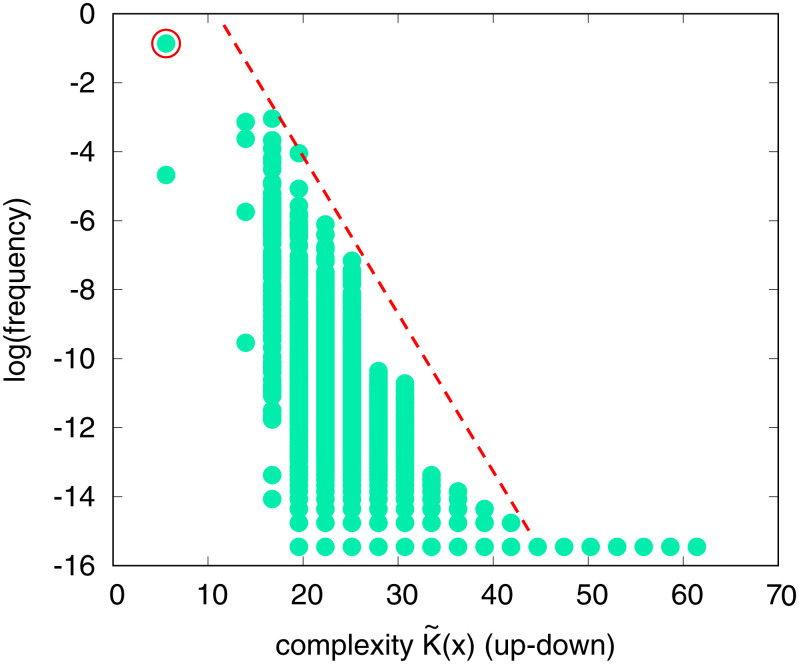
Scaled frequency vs. complexity $\tilde{K}(p)$ for the budding yeast ODE cell cycle model (30). Phenotypes are grouped by complexity of the time output of the key CLB2/SIC1 complex concentration. Higher frequency means a larger fraction of parameters generate this time curve. The red circle denotes the wild-type phenotype, which is one of the simplest and most likely phenotypes to appear. The dashed line shows a possible upper bound from Eq. **1**. There is a clear bias toward low-complexity outputs.

The protein and RNA phenotypes both describe shapes. Can a similar strong preference for simplicity be found for other classes of phenotypes? To answer this question, we also studied a celebrated model for the budding yeast cell cycle (30), where the interactions between the biomolecules that regulate the cell cycle are modeled by 60 coupled ordinary differential equations (ODEs). As a proxy for the genotypes, we randomly sample the 156 biochemical parameters of the ODEs (*Materials and Methods*). For each set of parameters, we calculate the complexity of the concentration versus time curve of the CLB2/SIC1 complex (a key part of the cycle) using the up–down method (31). Fig. 4 shows that *P*(*p*) exhibits an exponential bias toward low-complexity time curves, as hypothesized. Of course, many of these phenotypes may not supply the biological function needed for the budding yeast cell cycle. However, interestingly, the wild-type phenotype has the lowest complexity of all the phenotypes we found and is also the most likely to arise by random mutations. While the evolutionary origins of this GRN are complex, we again suggest that a bias toward simplicity in the arrival of variation played a key role in its emergence.

## Discussion

Our two main hypotheses are 1) upon random mutations, many GP maps are exponentially biased toward phenotypic variation with low descriptional complexity, as predicted by AIT (8), and 2) such strong bias in the arrival of variation can affect adaptive evolutionary dynamics, leading to a much higher prevalence of low-complexity (high-symmetry) phenotypes than can be explained by natural selection alone.

The arguments above are general enough to suggest that many biological systems, beyond the examples we provided, may favor simplicity and, where relevant, high symmetry, without requiring selective advantages for these features. For example, there are claims that model hydrophobic-polar (HP) lattice proteins with larger *P*(*p*) are typically more symmetric (32), and similar patterns have been suggested for protein tertiary structure in the PDB (33). In *SI Appendix*, section S6, we present further evidence that protein tertiary structure, signaling networks (34), and Boolean threshold models for GRNs (35) also exhibit bias in the arrival of variation. At a more macroscopic level, a model of tooth development (36) suggests that simpler phenotypes evolved earlier, consistent with a high encounter probability in evolutionary search. Similarly, for both teeth (37) and leaf shape (38), mutations to simpler tooth phenotypes are more likely than mutations to more complex phenotypes, an effect our theory also predicts. A recent theoretical study (39) of the development of morphology also found that simple morphologies were more likely to appear than complex ones upon random parameter choices. The L systems used to model plant development (4) show simplicity bias (8), and Azevedo et al. (40) showed that developmental pathways for cell lineages are significantly simpler (in a Kolmogorov complexity sense) than would be expected by chance.

Another interesting direction to investigate concerns the interplay of simplicity bias and logical depth (41), which measures the time it takes for a UTM to calculate the output from the shortest input strings. In this context, more complex GP maps are likely to have more logical depth, and the open question is how the effects we predict change for such systems.

At the other end of the spectrum, for complex phenotypic traits affected by many loci, variation may be more isotropic so that bias is weak. For such traits, where classical population genetics—which focuses on shifting allele frequencies in a gene pool where standing variation is abundant—typically works well, our arguments may no longer hold. The phenotype bias we discuss here is fundamentally about the origin of novel variation (19, 42) and so is most relevant on longer time scales.

Finally, simple phenotypes have a larger *P*(*p*) and are therefore more mutationally robust (1–3, 25, 43) (see also *SI Appendix*, section S4*B*). A correlation between low complexity and robustness is also found in the engineering literature (43, 44). Biological complexity often arises from connecting existing components together into modular wholes. If the individual components are more robust, then it is easier for them to evolve additional function, for example, a patch to bind to another protein, without compromising their core function. Similarly, a larger robustness may also enhance the ability of a system to encode cryptic variation, facilitating access to new phenotypes (45). Paradoxically, a natural tendency toward simpler and more robust structures may therefore facilitate the emergence of modularity, where individual components can evolve independently (46), and so make complex living systems more globally evolvable.

## Materials and Methods

### Protein Complex Topologies.

Our analysis of protein quaternary structure builds upon the techniques and data presented in ref. 16, where a curated set of 30,469 monomers, 28,860 homomers, and 5,527 heteromers were extracted from the PDB and classified into 120 distinct topologies. These were then used to make a periodic table of possible topologies. Protein complexes are described in terms of a weighted subunit interaction graph. An illustration of the topologies and how they are generated is shown in *SI Appendix*, Fig. S1, for two heteromeric complexes and their final graph topologies. Further examples of topologies and the PDB structures they describe can be found at http://www.periodicproteincomplexes.org/. The nodes of the graph are labeled according to their protein identities, and the weights of the connections are the interface sizes in Å^2^. The procedure for enumerating possible topologies and for classifying existing and potential topologies is described more fully in ref. 16. This approach only considers the largest interfaces, which if cut would disconnect the complex. The reason is that small interfaces that can be cut without disconnecting the complex are likely to be circumstantial and unlikely to play an important role in the assembly and evolution of the complex. After constructing the weighted subunit interaction graphs in this manner, we identify the topologically distinct interaction graph of subunit types (see, for example, *SI Appendix*, Fig. S1*C*, with the additional distinction between symmetric and asymmetric self-interactions of a subunit type, corresponding to homomeric interfaces.

We take the number of interface types of protein complex *p* to be the complexity measure $\tilde{K}(p)$. This choice scales with the number of individual mutations needed to generate the self-assembled complex. See *SI Appendix*, section S5*C*, for a longer discussion of different possible complexity measures. Unlike the polyomino case, where the building block is a square tile, the geometry of an individual protein is highly variable. For example, a cyclic homomeric 6-ring and a cyclic homomeric 10-ring will have the same topologically distinct interface configuration (which is just the two parts of the same asymmetric interface on a single subunit). This will be distinct from a heteromeric 6-ring in which we have two halves of two different symmetric interfaces on a subunit, and also distinct from a simple heterodimer. All three of these, however, have the same number of interface types (2) and so appear at $\tilde{K}(p)=2$ in the distribution of Figs. 1 and 2. The single point that appears at $\tilde{K}(p)=1$ for Fig. 2 is a homodimer, and the single point at $\tilde{K}(p)=0$ is a monomer. The symmetries of all protein complexes presented here are taken directly from the PDB.

To calculate the symmetries of all hypothetical protein complexes of size six in Fig. 1*C*, we used the following procedure. We first consider all topologically distinct graphs of size six with up to six different subunit types and symmetric or asymmetric homomeric interfaces between subunits of the same type. By comparing all 6! possible permutations of the adjacency matrix and the associated node labels we then calculate the permutation symmetries of the node types on these graphs as a proxy for the spatial symmetry of the hypothetical protein complexes that they represent. This collapses D3 and C6 into one category but allows us to distinguish this category from C3, C2, and C1 (and these from each other). Further discussion of the protein complexes can be found in *SI Appendix*, section S1.

### Polyominoes.

The polyomino model was implemented as described in refs. 6, 17, 18. The genome encodes a rule set consisting of 4*n* numbers which describe the interactions on each edge of *n* square tiles. Each number is represented as a length *b* binary string, so that the whole genome is a binary string of length L=4nb. The interactions bond irreversibly and with equal strength in unique pairs (1↔2, 3↔4, …), with types 0 and $2^{b}-1$ being neutral, not bonding to any other types. We label a given polyomino GP map with up to *n* possible tiles and 4*n* possible colors as $S_{n,4n}$; in this paper we usually work with $S_{16,64}$.

The assembly process is initiated by placing a single copy of the first-encoded subunit tile on an infinite grid. A different protocol where any tile may be used to seed the assembly is also possible and does not significantly affect the results presented here. Assembly then proceeds as follows. 1) Available moves are identified, consisting of an empty grid site, a particular tile, and a particular orientation, such that placing that tile in that orientation in the site will form a bond to an adjacent tile that has already been placed. 2) If there are no available moves, terminate assembly. 3) Choose a random available move, and place the given tile in that orientation at that site. 4) If the current structure has exceeded a given cutoff size, terminate assembly. 5) Go to step 1.

This process is repeated 20 times to ensure that assembly is deterministic—that is, that the same structure is produced each time. If different structures are produced or the structure exceeds a cutoff size (here taken to be larger than a 16 × 16 grid), the structure is placed in the category UND (unbounded or nondeterministic). For the calculation of probabilities/frequencies *P*(*p*) we ignore genotypes that produce the UND phenotype. This choice mimics the intuition that unbounded protein assemblies, or else proteins that do not robustly self-assemble into the same shape, are usually highly deleterious.

The rule set $S_{16,64}$ allows any 16-mer to be made since it is always possible to use addressable assembly where each tile is unique to a specific location. However, many 16-mers can be made with significantly fewer than 16 tile types, although there are examples that (to our knowledge) can only be made with all 16 tiles, so that a space allowing up to 16 tiles is needed.

To assign complexity values for the polyominoes, a measure similar to that used for the proteins was applied. First, the minimal complexity over the different genomes that generate polyomino *p* is estimated by sampling and finding the shortest rule set after removing redundant information. The search for a minimal complexity genome will be more accurate for high-probability polyominoes than for low-probability polyominoes. We checked that for most structures, only a fairly limited amount of sampling provided an accurate estimate of the minimal complexity; the minimal complexity genome is often the most likely to be found. The effects of finite sampling are illustrated further in *SI Appendix*, section S3 and Fig. S5. The complexity $\tilde{K}(p)$ of polyomino *p* is then given by the number of interfaces in the minimal genomes—thus, the number of genome regions encoding interfaces that play an active role in forming the final structure. A longer discussion of different choices of complexity measure, showing that the qualitative behavior is not very sensitive to details in the choice of approximate measure of algorithmic (Kolmogorov) information, can be found in *SI Appendix*, section S3*C*.

Evolutionary simulations of polyomino structures are performed following methods described in ref. 17: A population of *N* binary polyomino genomes is maintained at each time step. The assembly process is performed for each genome, and the resulting structure is recorded. UND genomes are assigned zero fitness. Other structures are assigned a fitness value based on the applied fitness function. These fitness values are used to perform roulette wheel selection, whereby a genome *g_i_* with fitness $f(g_{i})$ is selected with probability $f(g_{i})/\sum_{j}f(g_{j})$. Selection is performed *N* times (with replacement) to build the population for the next time step. Selected genomes are cloned to the next generation, then point mutations are applied with probability *μ* at each locus. A point mutation changes a 0 to a 1 and vice versa in the genome. We do not employ cross-over or elitism in these simulations.

We employ several different fitness functions. In the unit fitness protocol, all polyomino structures that are not UND are assigned fitness 1. In the random fitness protocol, each polyomino structure is assigned a fitness value uniformly randomly distributed on [0,1], and these values are reassigned for each individual evolutionary run. In the size fitness protocol, a polyomino of size *s* has fitness $1/(|s-s^{*}|+1)$, so that polyominoes of size $s^{*}$ have unit fitness and other sizes have fitness decreasing with distance from $s^{*}$. The simulations for Fig. 1*E* were done with *N* = 100 and μ=0.1 per genome, per generation. A number of other evolutionary parameters are compared in *SI Appendix*, section 3*C* and Fig. S6, showing that our main result—that the outcome of evolutionary dynamics exhibit an exponential bias toward simple structures—is not very sensitive to details such as mutation rate or the choice of fitness function.

### RNA Secondary Structure GP Map.

For *L* = 30 RNA, we randomly generated 32,000 sequences, and for *L* = 100, we generated 100,000 random sequences. As in refs. 25, 26, secondary structure (SS) is computationally predicted using the fold routine of the Vienna package (23) based on standard thermodynamics of folding. All folding was performed with parameters set to their default values (in particular, the temperature is set at $T=37^{{}^{\circ}}$ C). We then calculated the neutral set size [NSS(p)], the number of sequences mapping to a SS *p*, for each SS found by random sampling, by using the neutral set size estimator described in ref. 27, which is known to be quite accurate for larger NSS structures (25). We used default settings except for the total number of measurements (set with the -m option), which we set to 1 instead of the default 10, for the sake of speed, but this does not noticeably affect the outcomes we present here.

RNA structures can be represented in standard dot-bracket notation, where brackets denote bonds and dots denote unbonded pairs. For example, …((…))…. means that the first three bases are not bonded, the fourth and fifth are bonded, the sixth through ninth are unbonded, the tenth base is bonded to the fifth base, the eleventh base is bonded to the fourth base, and the final four bases are unbonded. For shorter strands such as *L* = 30, the same SS can be found multiple times in the fRNAdb.

For longer strands, finding multiple examples of the same SS becomes more rare, so that SS frequencies cannot be easily directly extracted from the fRNAdb. However, it seems reasonable, especially for larger structures, that fine details of the structures are not as important as certain more gross structural features that are captured by a more coarse-grained picture of the structure. In this spirit, we make use of the well-known RNA abstract shape method (29) where the dot-bracket SS are abstracted to one of five hierarchical levels, of increasing abstraction, by ignoring details such as the length of loops but including broad shape features. For the *L* = 100 data we choose the fifth or highest level of abstraction which only measures the stem arrangement. This choice of level is needed to achieve multiple examples of the same structure in the fRNAdb database, so that a frequency can be directly determined with statistical significance. The SS were converted to abstract shapes with the online tool available at https://bibiserv.cebitec.uni-bielefeld.de/rnashapes. Using these coarse-grained structures means that the theoretical probability *P*(*p*) can be directly calculated from random sampling of sequences, where *N_G_* is the number of sequences, which for an RNA GP map for length *L* RNA is given by $N_{G}=4^{L}$. A similar calculation of the *P*(*p*) for RNA structures for *L* from 40 to 126 at different levels of coarse-graining can be found in ref. 26.

To generate the distributions of natural RNA we took all available sequences of *L* = 30 and L= 100 from the noncoding fRNAdb (28). As in ref. 25, we removed a small fraction (∼1%) of the natural RNA sequences containing nonstandard nucleotide letters, e.g., N or R, because the standard folding packages cannot treat them. Similarly, a small fraction (∼2%) of sequences were also discarded due to the neutral set size estimator failing to calculate the NSS (this is only relevant for *L* = 30). We have further checked that removing by hand any sequences that were assigned putative roles, or are clear repeats, does not significantly affect the strong correlation between the frequencies found in the fRNA database and those obtained upon random sampling of genotypes. For a further discussion of the question of how well frequency in the databases tracks the frequency in nature, see also refs. 25, 26 and *SI Appendix*, Fig. S10, where a comparison with the Rfam database is also made. Note that the similar behavior we find across structure prediction methods, strand lengths, and databases would be extremely odd if artificial biases were strong on average in the fRNA database. We used 40,554 unique RNA sequences of *L* = 30, taken from the fRNAdb, corresponding to 17,603 unique dot-bracket structures. Similarly, we used 932 unique fRNAdb *L* = 100 RNA sequences, corresponding to 17 unique level 5 abstract structures/shapes.

To estimate the complexity of an RNA SS, we first converted the dot-bracket representation of the structure into a binary string *p* and then used the Lempel–Ziv-based complexity measure from ref. 8 to estimate its complexity. To convert to binary strings, we replaced each dot with the bits 00, each left bracket with the bits 10, and each right bracket with 01. Thus, an RNA SS of length *n* becomes a bit string of length 2*n*. Because level 5 abstraction only contains left and right brackets, i.e., [and], we simply convert the left bracket to 0 and the right to 1 before estimating the complexity of the resulting bit string via the Lempel–Ziv-based complexity measure from ref. 8. The level 5 abstract trivial shape with no bonds is written as underscore, and this we simply represented as a single 0 bit. *SI Appendix*, section S4 provides more background on RNA structures, and *SI Appendix*, section S5*B* provides more detail of the complexity measure.

### GRN of Budding Yeast Cell Cycle.

The budding yeast (*Saccharomyces cerevisiae*) cell cycle GRN system from ref. 30 consists of 60 coupled ODEs relating 156 biochemical parameters. The model parameter space (i.e., genotype space) was sampled by picking random values for each of the parameters by multiplying the wild-type value by one of {0.25,0.50,…,1.75,2.00}, chosen with uniform probability. The ODEs generate concentration–time curves for different biochemicals involved in cell cycle regulations. All runs were first simulated for 1,000 time steps, with every time step corresponding to 1 min. Next, we identified the period of every run (usually on the order of 90 time steps), took one full oscillation, and coarse-grained it to 50 time steps. This way, if two genotypes produce curves which are identical up to changes in period, they should ultimately produce identical or nearly identical time series and binary string phenotypes. For every genotype or set of parameters, the curves for the CLB2/SIC1 complex are then discretized into binary strings using the up–down method (31): for every discrete value of t=δt,2δt,3δt,…, we calculate the slope *dy*/*dt* of the concentration curve, and if dy/dt≥0, a 1 gets assigned to the *j*th bit of the output string; otherwise, a 0 is assigned to it. All strings with the same up–down profile were classified as one phenotype. To generate the *P*(*p*) in Fig. 4, $5\times 10^{6}$ inputs were sampled. Complexity $\tilde{K}(p)$ is assigned by using the Lempel–Ziv measure from ref. 8 (see also *SI Appendix*, section S5*B*) applied to binary output strings. As shown in ref. 8, this methodology works well for coupled differential equations, and the choice of input discretization, sample size, and initial conditions does not qualitatively affect the probability–complexity relationships obtained. The wild-type curve can be observed in in figure 2 of ref. 30, where it is labeled Clb2*_T_*.

## Supplementary Material

Supplementary File

## Data Availability

All study data are included in the article and/or *SI Appendix*.

## Change History

April 11, 2022: The article license has been updated.
