# Supplementary material for: Symmetry and simplicity spontaneously emerge from the algorithmic nature of evolution
Source: Proc Natl Acad Sci U S A. 2022 Mar 11;119(11):e2113883119. doi: 10.1073/pnas.2113883119 (PMC8931234; doi:10.1073/pnas.2113883119)
Supplement: Supplementary File [file pnas.2113883119.sapp.pdf]

Supporting Information for:
Symmetry and simplicity spontaneously emerge from the algorithmic nature of evolution

Iain G Johnston [a,b,c,d], Kamaludin Dingle [e], Sam F. Greenbury [f,g] Chico Q. Camargo [h] Jonathan P. K. Doye [i] Sebastian E. Ahnert [d,f,j] and Ard A. Louis [c,1]

[a]Department of Mathematics University of Bergen; Norway. [b]Computational Biology Unit, University of Bergen, Bergen; Norway [c]Rudolf Peierls Centre for Theoretical Physics, University of Oxford; Oxford, United Kingdom. [d]The Alan Turing Institute, British Library; London, United Kingdom. [e]Centre for Applied Mathematics and Bioinformatics, Department of Mathematics and Natural Sciences, Gulf University for Science and Technology; Kuwait. [f]Theory of Condensed Matter Group, Cavendish Laboratory, University of Cambridge; Cambridge, United Kingdom [g]Department of Metabolism, Digestion and Reproduction, Imperial College London; London, United Kingdom [h]Dept of Computer Science, University of Exeter; Exeter, United Kingdom. [i]Physical and Theoretical Chemistry Laboratory, Department of Chemistry, University of Oxford; Oxford, United Kingdom. [j]Department of Chemical Engineering and Biotechnology, University of Cambridge; Cambridge, United Kingdom.
[1]To whom correspondence should be addressed; E-mail:ard.louis@physics.ox.ac.uk.

This Supporting Information File includes:

Supplementary Text
Fig S1 – S17
Table I – III
References

S1. SUPPLEMENTARY TEXT FOR PROTEIN QUATERNARY STRUCTURE

While the tertiary structure of a protein describes the folded state of an individual polypeptide chain, the quaternary structure of a protein describes how individual protein subunits bond to form the final complex. Over half the proteins found in nature (so far) form homomeric or heteromeric complexes with other proteins, and these structures tend to be highly conserved on evolutionary time-scales [2, 3]. Interestingly, the physical assembly pathways may mimic the evolutionary pathways that led to a particular protein assembly [4, 5]. In ref. [1] the authors combined bioinformatic searches together with electrosplay mass spectrometry experiments to hypothesise that protein complex topologies can be generated from combinations of three basic types of assembly steps, namely dimerisation, cyclisation, and heteromeric sub-unit addition. These combinations were used to generate a periodic table of (theoretically) possible topologies. Most protein complex topologies found in the PDB were shown to fall into one of the topologies predicted from their procedure, and thus can be classified into their periodic table. At the same time, many potential topologies have not (yet) been found. Most of these are complex, that is they would need a larger complexity $\tilde{K}(p)$ to be produced than the values we measure for existing structures. This fact is consistent with our hypothesis that complex topologies are harder to evolve than simpler ones are, and so less likely to appear in nature. In Fig. S1, we illustrate how the methods from ref. [1] are used to generate topologies and complexities of the proteins structures.

A key question is whether or not the patterns we observed in the PDB mirror patterns seen more widely in the biological world. While this question is hard to answer *a priori*, we note that it would be extremely surprising if the strong bias towards symmetry (and low descriptional complexity) were merely an artefact of the PDB. To further investigate this question, we follow methodology from ref. [1] by applying a stringent redundancy filter that removes multiple copies of the same protein from the data if they share more than 50% sequence identity. This filter is used for a subset of the analysis conducted in [1], and the filtered subset of complexes is provided as part of the Supplementary Information of that publication. As can be seen when comparing Fig. S2 (A) to Fig. 1B in the main text, this procedure reduces the amount of data significantly from 1778 to 545 6-mer topologies, but the overall trend is very similar, supporting our hypothesis that the global bias towards symmetry observed in the PDB mirrors a broader trend in nature, and is not a database artefact.

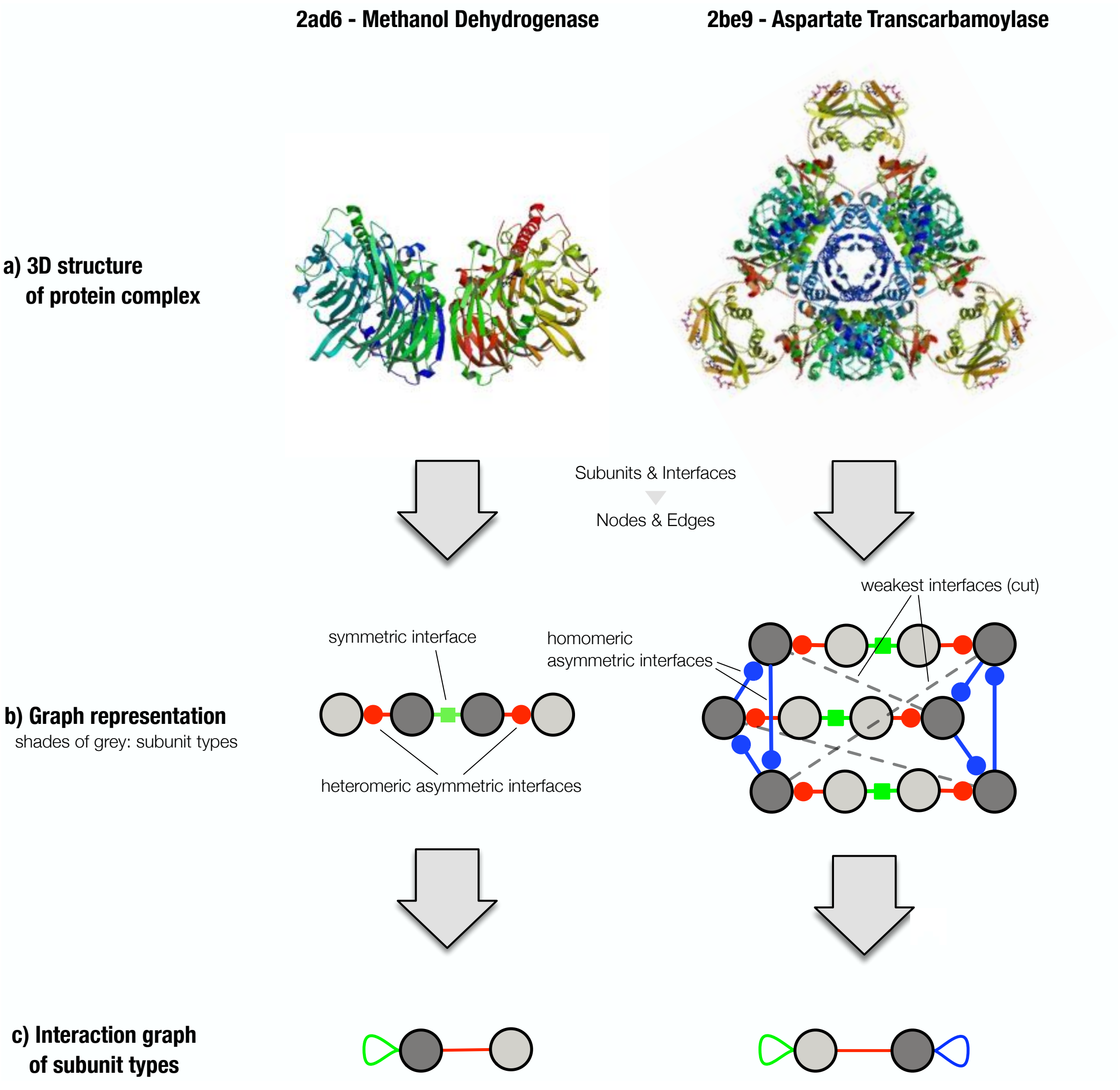

FIG. S1. **Graph representation of protein-complex topologies following the methods from ref. [1]** (a) Two heteromeric complexes, a 4-mer methanol dehydrogenase (2ad6), and a 12-mer aspartate transcarbamoylase (2be9). (b) Symbolic graph representations of the proteins and the interfaces between them. Different shades of grey denote different types of protein subunit (according to sequence similarity). Interface colours denote symmetric (green), homomeric asymmetric (blue), and heteromeric asymmetric (red) interlaces. Weak interfaces that are disregarded (see Methods section IA) are shown as dashed lines. (c) Interaction graph of subunit types, which is a reduced representation of the same complex that shows each type of distinct interface that exists in the complex exactly once. For instance, the green self-loop on the light grey node in the left column indicates the symmetric (green) interaction with another copy of the same subunit type in b), whereas the red connection denotes the heteromeric interaction between the two different subunit types. The complexity of a protein complex is given by the number of interface types, where symmetric interfaces (green) consist of one self-interacting type, and asymmetric interfaces (blue and red) consist of two types. The complexities of the examples shown are therefore $\tilde{K}(p) = 3$ for 2ad6 and $\tilde{K}(p) = 5$ for 2b39.

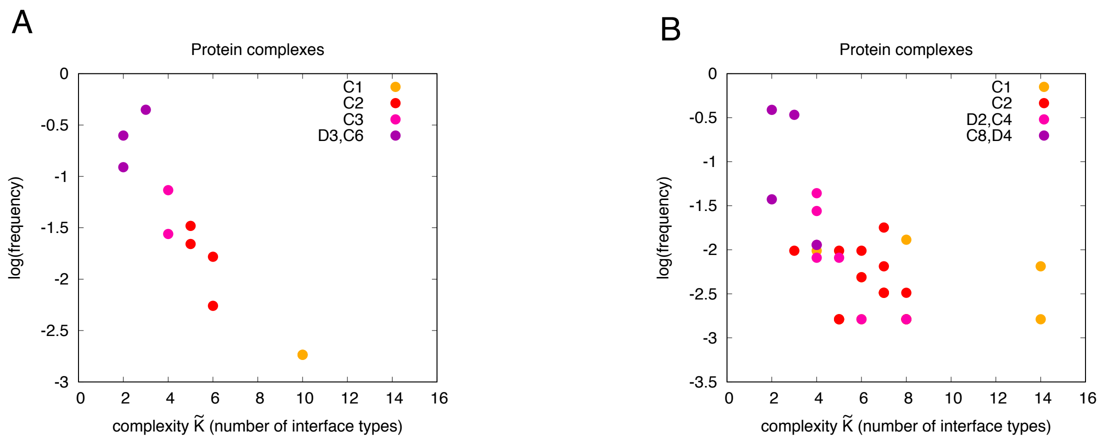

FIG. S2. **(A)** Frequency of 6-mer protein complex topologies found in the PDB versus the number of interface types (a measure of complexity), as in Fig. 1B of the main text, but with a redundancy filter from [1] applied. **(B)** Frequency of 8-mer protein complex topologies versus complexity (no redundancy filter). For both sizes there is a strong preference for low complexity/high symmetry structures. Symmetries are in standard Schoenflies notation.

The main text only shows data for 6-mers. We found similar trends for other sizes. As an example, in Fig, S2 (B) we show a frequency vs. complexity plot for 8-mers, which resembles results for other sizes as well.

In Fig. 1 of the main text we showed that structures found in the PDB were highly biased towards a small subset of highly symmetric structures. In Fig. S3A we show the 6-mer topologies in more detail, including their symmetries. This plot helps illustrate the main point observed in Fig. 1 of the main text, which is that highly symmetric structures are much more likely to appear in nature than structures with lower symmetry.

Fig. S3B is similar in spirit to Fig. S3A in that it complements Fig. 1 for polyominoes. It helps illustrate how extremely strong the bias in the arrival of variation towards high symmetry structures is. Note that in this evolutionary run, all 16mers have the same fitness.

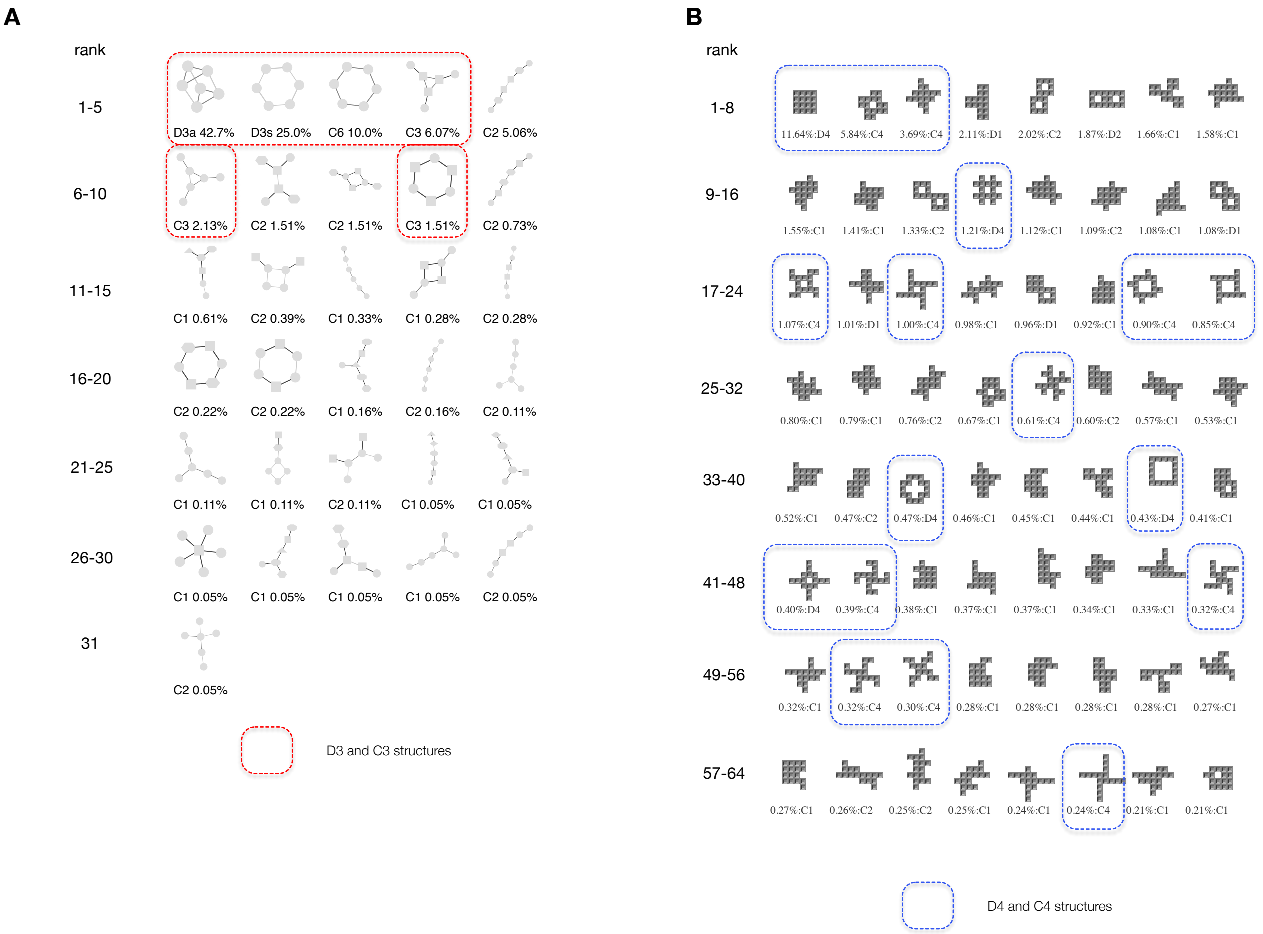

FIG. S3. **A) Protein 6-mer complex topologies from Fig 1B, ordered by their frequency in the PDB database**. The highlighted ones are those with symmetry C_3 or higher. The higher symmetry ones are significantly more likely, on average, to appear in the database than lower symmetry structures. Similar results are found for other sizes. Darker and paler lines corresponds to asymmetric and symmetric interfaces, respectively. **B) Most frequently found 16-mer polyomino structures from Fig 1E**. These 64 most likely structures (out of 13,079,234 possible 16-mers) include all 5 of the most symmetric polyominoes with D_4 symmetry, and all 12 with the next symmetry (C_4). Together these 64 structures make up about 64% of the total probability weight for this evolutionary run even though they only make up a tiny fraction $\approx 1/200,000$ of all possible structures. By contrast, while the low symmetry C_1 structures make up 99.9% of all possible polyomino shapes, they are heavily suppressed in the evolutionary run relative to higher symmetry shapes. All 16-mer structures share the same fitness, so while the emergence of a 16-mer is due to natural selection, the strong bias towards simpler/more symmetric structures illustrated here is not caused by natural selection, but rather by the very large differences in rates of the arrival of variation.

S2. POPULATION DYNAMICS AND ARRIVAL OF THE FREQUENT FOR A HIGHLY BIASED GP MAP

A. Basic introduction to the arrival of the frequent

Fig. 1 from the main text shows the results of an evolutionary simulation for polyominoes with a fitness function that has a maximum for size $s^* = 16$. By invoking the "arrival of the frequent" population dynamics framework from ref. [6], we argue that, for a wide range of population dynamic parameters, the frequencies with which phenotypes occur upon averaging over evolutionary runs should be qualitatively similar to the probabilities $P(p)$ that they are obtained upon random sampling over sequences. From the perspective of traditional population genetics, where bias in the introduction of variation is rarely taken into account [7, 8], it may seem highly surprising that random sampling over genotypes should be able to make such predictions when a GP map is highly biased. We therefore briefly sketch out some key arguments from the arrival of the frequent framework below.

The first aspect of this framework to note is that given a genotype that maps to a phenotype y, the *mean* probability ϕ_{py} (averaged over the whole neutral network of all genotypes mapping to phenotype y) that novel phenotype $p \neq y$ arises by a random point mutation can, to first order, be approximated quite well by the *global* probability $P(p)$ that p appears upon random

sampling of genotypes, regardless of the source phenotype y. This relationship has been observed for a range of GP maps [6, 9–11]. We show explicitly in Fig. S6C that this prediction of global frequency being a good predictor of the average rate at which a phenotype arises in an evolving population works remarkably well for evolutionary polyomino simulations. In [6] the same effect is demonstrated for simulations of the $L = 20$ RNA map. While there will obviously also be deviations from such a simple rule, and these are indeed observed [6, 9–11], when the frequencies vary by many orders of magnitude, as they do for the GP maps studied here (and as the AIT formalism predicts will be true much more widely [12]) then these deviations are relatively small on the scale of the range of possible frequencies. If one is interested in predictions on these larger scales, then the global frequency of a phenotype will be a good first order guide to the rate at which that particular phenotype variation arises in a population. The argument above about the equivalence of local and global frequencies is crucial for understanding why we can take such a simple random sampling of sequences, and yet make predictions for the probabilities of outcomes that may have arisen from many complex evolutionary histories.

Interestingly, and in contrast to the probability ϕ_{py} with $p \neq y$ that new phenotypes appear upon mutations in a population, the mutational robustness $\rho_y = \phi_{yy}$, defined as the mean probability that a point mutation generates the same phenotype as the source y, shows much less variation, and scales as $\rho_y \sim \log(P(y))$ for all the GP maps that we are aware of [6, 9, 10, 13–16]. Since $P(p)$ can vary over many orders of magnitude, the probability that a particular novel phenotype arises by mutations can vary by many orders of magnitude, depending to first order on its $P(p)$. By contrast, the robustness ρ varies much less dramatically for a typical GP map.

Next consider a population with a carrying capacity of N individuals, a mutation rate of μ per site and a genome length of L. Then as shown in ref. [6], under the assumptions above, which hugely simplify the population genetic equations, the average over evolutionary runs of the median time $T_{\frac{1}{2}}(p)$ to discover a particular new phenotype p is well described by the simple analytic form

$$T_{\frac{1}{2}}(p) \approx \frac{\log(2)}{NL\mu P(p)}. \tag{1}$$

in polymorphic limit ($NL\mu \gg 1$), and by

$$T_{\frac{1}{2}}(p) \approx \frac{\log(2)}{L^2(K-1)\mu\rho P(p)} \tag{2}$$

in the opposite monomorphic limit where $NL\mu \ll 1$. Here K is the alphabet size of the genome, and the robustness ρ is that of the neutral network the system is on. Extensive evolutionary simulations for a range of populations sizes and mutation rates showed that both these analytic forms quantitatively describe the discovery time $T_{\frac{1}{2}}(p)$ [6] in their respective regimes, and that one can smoothly interpolate between them for intermediate regimes.

The AIT upper bound on $P(p)$, (Eq. (1) in the main text and Eq. (17) in this SM, allows us to derive lower bounds for the median discovery time in the polymorphic regime:

$$T_{\frac{1}{2}}(p) \gtrsim \frac{\log(2)}{NL\mu\, 2^{-a\tilde{K}(p)+b}}. \tag{3}$$

and in the monomorphic regime:

$$T_{\frac{1}{2}}(p) \gtrsim \frac{\log(2)}{L^2(K-1)\mu\rho\, 2^{-a\tilde{K}(p)+b}}, \tag{4}$$

where we have ignored for now the scaling of ρ with the phenotype the system starts from, since this typically varies by less than an order of magnitude for phenotypes of interest, much less than the many orders of magnitude variation in $P(p)$.

The observed variation in $P(p)$ (or $\tilde{K}(p)$) immediately implies that the discovery time $T_{\frac{1}{2}}(p)$ of a new phenotype p will typically vary over many orders of magnitude, with simple phenotypes being exponentially more likely to occur than more complex phenotypes. Since evolutionary time-scales for real populations are limited, typically only the most frequent phenotypes are likely to arise as potential variation for natural selection to act on. And thus it is only these frequent phenotypes that can fix, and that are therefore observed in nature. This *arrival of the frequent* effect [6] has already been observed for non-coding functional RNA where only a minescule fraction of all possible SS are observed in nature. Indeed those that are found can be shown to be a small set of the most frequent structures with relatively large NSS or $P(p)$ [17, 18].

We note that this effect differs fundamentally from mechanisms such as the *survival of the flattest* [19], which implicitly assume that frequent and rare phenotypes are both present as potential variation. By contrast, the arrival of the frequent is fundamentally a non-ergodic and time-dependent effect, it acts as an effective constraint preventing many phenotypes from ever appearing as potential variation. It is not a steady-state effect such as the survival of the flattest. In the limit of infinite time all potential variation could appear, and then effects such as the survival of the flattest could be more relevant for (see [6] for a discussion). However, that regime is not (even nearly) reached for the systems we study.

In summary, within this arrival of the frequent picture, very strong biases in $P(p)$ translate into equivalent strong biases in median discovery times for phenotypes, $T_{\frac{1}{2}}(p)$, which, in turn, play an important role in determining evolutionary outcomes. When the range of $P(p)$ is large, as we argue is generically the case for the GP maps of protein quaternary structure, RNA SS, and GRN that we study, then the arrival of the frequent effect may in fact dominate (on average) over detailed selective pressures, and sto strongly affect adaptive evolutionary dynamics. See also refs. [7, 20] for a much broader discussion of the rate of introduction of new phenotypes in the evolutionary literature, and [21] for an important discussion of bias in the introduction of new variation.

In Fig. S4(a) we show some typical trajectories for a simulation with a fitness function that is maximal for 16-mers. In Fig. S4(b) we schematically illustrate the main idea of the arrival of the frequent [6].

In section S3 C we use evolutionary simulations to illustrate how the arrival of the frequent mechanism described above determines evolutionary outcomes for a range of population dynamic parameters, and different fitness functions. When this effect dominates, the outcomes only weakly depend on the details of the evolutionary dynamics. From experience we are aware that this result may seem highly surprising to some, but the data we present here strongly supports our arrival of the frequent picture.

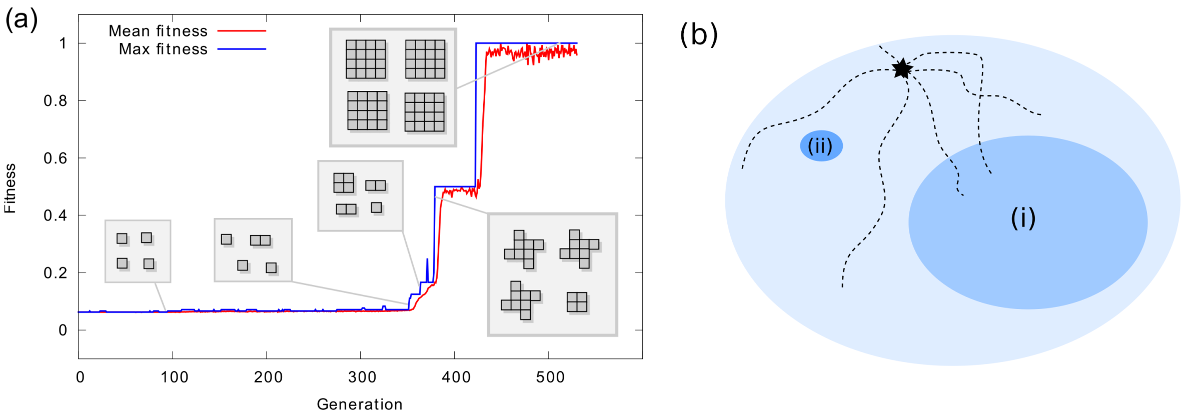

FIG. S4. **Schematic picture of evolutionary trajectories in search space.** (a) An example of a typical evolutionary simulation run in the directed evolution model for $s^* = 16$ for $N = 100$ and $\mu = 0.1$. Traces show mean and maximal fitness within a population, insets show a snapshot of a subset of the population at a given time. Starting from a population of simple structures, more complex structures evolve as evolutionary innovation discovers bonding approaches to produce fitter (larger) polyominoes. (b) A schematic figure illustrating how, if a population starts at phenotype (star), it is much more likely to find a phenotype such as **i** with a large NSS, than a phenotype such as **ii** which has a small NSS, even if the latter is more fit than the former. In the case of (a) and Fig. 1 in the main text, all 13,079,255 16-mers have the same maximum fitness, but a structure such as the square is much more likely to appear than a low symmetry structure because the square has a large NSS.

B. Arrival of the frequent and the analogy between evolution on biased GP maps and deep learning

There is an interesting analogy between the arrival of the frequent in a highly biased evolutionary system, and training deep neural networks (DNNs) in the context of supervised learning [22]. Given a training set $S = \{x_i, y_i\}$ of input-output pairs, where the x_i are drawn from an input space $\mathcal{X}$, and the y_i are drawn from an output space $\mathcal{Y}$, the supervised learning training task is to find a set of neural network parameters such that the DNN, upon the set of inputs $\{x_i\} \in S$, produces a set of outputs $\{\hat{y}_i\}$ that minimises a loss function $L(y, \hat{y})$. Zero training error means that $y_i = \hat{y}_i$ for all $y_i \in S$. The most popular optimisation technique to find parameters that minimise the loss function is called stochastic gradient descent (SGD). This method follows the local gradient of the loss landscape. This problem of minimizing a loss-function by using local information is similar to an evolutionary system which maximises a fitness function. In fact, genetic algorithms, inspired by evolutionary dynamics, have been used to train DNNs, see e.g. [23] for a recent application for supervised learning, or [24] for an application of genetic algorithms to DNN based reinforcement learning.

DNNs can be viewed as function approximators, where the function f determines which outputs $\{\hat{y}_i\}$ the DNN produces upon inputs $\{x_i\}$. A parameter-function map can be defined as follows. The space of functions the model can express is a set

$\mathcal{F} \subseteq \mathcal{Y}^{|\mathcal{X}|}$. If the model takes parameters within a set $W \subseteq \mathbb{R}^n$, then the parameter-function map $\mathcal{M}$ is defined as

$$\mathcal{M} : W \to \mathcal{F}$$
$$\mathbf{w} \mapsto f(\mathbf{w}).$$

Since DNNs are typically employed in the overparameterized regime, with many more parameters than inputs or outputs, there are typically many different sets of parameters that map to the same function f. One can define a probability $P(f)$ that a DNN produces a function f upon random sampling of its parameters (for simplicity, consider functions f restricted to the domain defined by the set of inputs $\{x_i\}$ of interest.). It has been recently shown [25] that, just as for GP maps, this parameter-to-function map follows the same AIT predicted simplicity bias scaling from Eq. (17). In other words, DNNs have a strong bias towards simple functions upon random sampling of parameters.

At first sight, the relevance of $P(f)$ may not be immediately obvious, since DNNs are not trained by random sampling of parameters, which would be very inefficient, but rather by SGD or related optimisation techniques which start with a particular set of parameters, and make local moves in parameter space to reach a loss minimum (in direct analogy to finding a (local) fitness maximum for evolutionary systems). Interestingly, it has recently been shown for a wide range of data sets and for different loss functions, that training by SGD, which only takes into account local information on the loss function $L(y, \hat{y})$, when averaged over initial conditions, generates functions f with a probability $P_{SGD}(f) \approx P(f)$ [26]. This behaviour has as strong resemblance with what we find in this paper for evolutionary systems, where the global probability $P(p)$ that a particular phenotype p appears upon random sampling is predictive of an evolutionary process that only locally samples a fitness function. Both the DNN parameter-function map, and the evolutionary GP maps, are highly biased. For such highly biased systems, the arrival of the frequent, or closely related phenomenology, explains why random sampling of parameters predicts outcomes for a wide range of different detailed dynamical histories. The fact that we see similar phenomenology in DNNs and in GP maps, suggests that the prediction that a local optimiser may still, on average, generate outputs with probability given by the global probability, may hold more widely for highly biased input-output maps. Thus these empirical results from DNNs support our arrival of the frequent picture in GP maps.

S3. SUPPLEMENTARY TEXT FOR POLYOMINOES

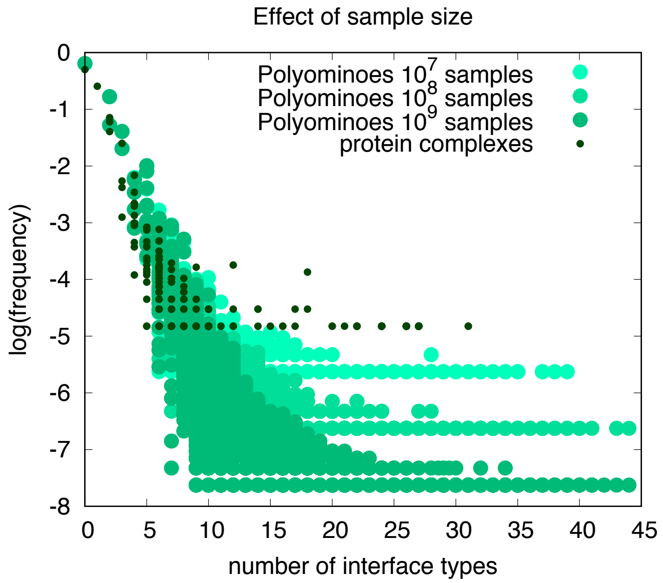

FIG. S5. Frequency with which a polyomino p appears as a function of complexity $\tilde{K}(p)$= number of interfaces, for random sampling of genotypes with different numbers of samples. The plot illustrates how the long tail at lower occurrence probability $P(p)$ with high complexity is caused by finite sampling effects. In addition, the plot shows the frequency that a protein complex topology p is found in the PDB as a function of $\tilde{K}(p)$, for all 34,287 topologies in the 3DComplex database (black dots). We conjecture that, just as found for polyominoes, this tail will also reduce for proteins as more topologies are published in the PDB. Nevertheless, there are likely also specific adaptive causes for certain of the low frequency/high complexity protein complex structures that may therefore have higher probability than expected from their complexity.

A. Effects of finite sampling of genotype space on polyominoes

To test the effect of finite sampling on the $P(p)$ versus $\tilde{K}(p)$ plot for polyominoes, we show in Fig. S5 data for 10^7, 10^8 and 10^9 samples on $S_{4,16}$, (4 tile types, 16 patch types). The main effect of a weaker sampling protocol is the emergence of a heavy tail at low frequency ($P(p)$) and high $\tilde{K}(p)$. There are two reasons for this tail. The first is that we estimate $\tilde{K}(p)$ from the simplest ruleset found in the sampling that generates the polyomino, and so fewer samples means a smaller likelihood that we find the simplest ruleset, resulting in a higher apparent complexity. However, because the simplest rulesets are typically the most likely ones to be found, we expect the error here to typically be relatively small, even for structures that are rare. The second and more important reason for the long tail arises simply from poor sampling of $P(p)$. A low $P(p)$ structure may occur by chance just once or twice, and so be assigned a much higher frequency than if more sampling is done. This second source of error was also identified in ref. [12] (see e.g. Fig. 9 in the Supplementary Information of that paper, which shows the same tails for a circadian rhythm model where the complexities can be directly defined without sampling, so that only the second sampling argument causes the tails.). All this evidence suggests that the main source of the long tails is the poor sampling of $P(p)$, and not errors in the measurements of $\tilde{K}(p)$.

We note that Fig. S5 also shows a large tail for the protein complex topologies. Part of the cause of this tail may also be the relatively low sampling, as found above for the polyominoes. However, it is also likely that some rare high complexity structures are the result of specific adaptive causes that favour larger sizes and complexities. It would be very interesting to see how these trends change as more structures are deposited into the PDB.

B. Calculating neutral space sizes in the polyomino model

In this section we analytically explore the link between the structure of a minimal genome required to produce a polyomino structure (and hence its complexity) and that structure's neutral space space. We proceed as follows: Consider a genome G of length L that encodes a phenotype (polyomino topology) p. If s specified bonds appear in the reduced genome representation of G (i.e. with all non-bonding edges set to a neutral label), then $l = L - s$ elements of G are, to some extent, unconstrained. We may pick interaction labels to occupy these sites as long as these labels do not bond to the specified elements or form bonds among themselves (and thus perturb the desired structure). In this combinatoric analysis, we first calculate the number of ways of choosing a given number of labels to fill the available genome sites, so that those labels do not interfere with bonding. We then consider the ordering of these labels, and finally the orderings of the bonding information in the genome.

Consider genomes consisting of an alphabet of n_c labels, n_0 of which are neutral with respect to every other bond. Denote by b the number of bonding pairs in a reduced genome, so that $n_p = n_c - n_0 - 2b$ labels remain unused in the reduced genome and bond to one other partner. We will proceed by considering the number of ways we can populate the l unconstrained sites in a genome with neutral labels, interacting labels that do not interfere with bonding, or a combination of both. The number of ways of picking a set of exactly i distinct neutral labels is

$$n_{\text{neutral}}(i) = \frac{1}{i!} \frac{n_0!}{(n_0 - i)!}, \tag{5}$$

where, for later convenience, the prefactor ensures that different orderings of the set do not contribute to the total. If we choose a label that interacts with a partner to fill a neutral space, we must ensure that we do not pick the partner at any point in building our neutral string. With this constraint, the number of sets of exactly i bonding labels that are effectively neutral, i.e. are not able to form any bonds due to the absence of partners, is

$$n_{\text{nonbonding}}(i) = \frac{1}{i!} \frac{n_p!!}{(n_p - 2i)!!} \tag{6}$$

Then, the total number of ways of choosing exactly c distinct non-interfering labels is the sum over i of the ways of picking i neutral and $c - i$ non-interfering but partnered labels:

$$n_{\text{non-interfering}}(c) = \sum_{i=0}^{c} n_{\text{neutral}}(i) n_{\text{nonbonding}}(c - i). \tag{7}$$

We now consider the different orderings of these labels within the available sites. The number of words of length l containing exactly c distinct characters is defined by the recursive expression

$$n_{\text{words}}(l, c) = c^l - \sum_{i=1}^{c-1} \binom{l}{i} n_{\text{words}}(l, i) \tag{8}$$

$$n_{\text{words}}(l, 1) = 1. \tag{9}$$

This expression equates the number of words of length l with exactly c characters to the total number of words of length l with at most c characters (c^l), with all possible words with fewer than c characters subtracted from this total. As we ordered our sets of available colours, double counting of identical sequences is prevented. So an expression for the neutral space size consists of a combination of the above expressions for (a) ways of picking c distinct non-interfering colours and (b) ways of arranging these c colours in l spaces:

$$r(l|n_0, n_p) = \sum_{j=1}^{n_p/2+n_0} \left(\sum_{i=0}^{j} n_{\text{neutral}}(i) n_{\text{nonbonding}}(j-i) \right) n_{\text{words}}(l, j). \tag{10}$$

This expression gives the neutral space associated with a particular reduced genome.

As an example, consider the situation in which we have $l = 2$ spaces, $n_0 = 2$ neutral labels, and $n_p = 2$ unused bonding labels. If the neutral labels are `0` and `7` (as in $\mathcal{S}_{2,8}$), and the unused bonding labels are `5` and `6`, we can populate the empty spaces through the following:

- Use only one label to populate the two spaces ($n_{\text{words}}(2, 1)$):
  - Use a neutral label:
    $n_{\text{neutral}}(1) n_{\text{nonbonding}}(0) n_{\text{words}}(2, 1) = 2$: (`00`, `77`)
  - Use a bonding label:
    $n_{\text{neutral}}(0) n_{\text{nonbonding}}(1) n_{\text{words}}(2, 1)$: (`55`, `66`)
- Use two labels to populate the two spaces ($n_{\text{words}}(2, 2)$):
  - Use only neutral labels:
    $n_{\text{neutral}}(2) n_{\text{nonbonding}}(0) n_{\text{words}}(2, 2) = 2$: (`07`, `70`)
  - Use a neutral and a bonding label:
    $n_{\text{neutral}}(1) n_{\text{nonbonding}}(1) n_{\text{words}}(2, 2) = 8$: (`05`, `06`, `75`, `76`, `50`, `60`, `75`, `76`)
  - Use only bonding labels:
    $n_{\text{neutral}}(0) n_{\text{nonbonding}}(2) n_{\text{words}}(2, 2) = 0$ (as `56` would form a new bond)

So overall $r(2|2, 2) = 14$.

In general, there are also transformations to the reduced genome that leave the phenotype invariant.

1. Recalling that b denotes the number of bonding pairs involved in the reduced genome, the labels of bonds can be swapped for each pair (e.g. `3400` ↔ `4300`), giving

$$s_1 = 2^b \tag{11}$$

invariant genomes.

2. Any of the available bonding pairs can be used in place of any other (e.g. `3400` ↔ `5600`), giving

$$s_2 = \frac{n_p/2!}{(n_p/2 - b)!} \tag{12}$$

genomes.

3. Bonds can be rotated around a given subunit specification (e.g. `3400` ↔ `0340`), giving

$$s_3 = \prod_i n_{\text{rotations}}(B_i) \tag{13}$$

genomes, where $n_3(B_i)$ is the number of invariant rotations that can be made on block B_i that are not degenerate with other transformations. For example, $n_{\text{rotations}}(\texttt{1111}) = 1$, $n_{\text{rotations}}(\texttt{1112}) = 4$. $n_{\text{rotations}}(\texttt{1122}) = 2$, as the arrangement `1221` cannot be reached by other transformations, but `2211` and `2112` can be reached by the pair-swapping transformation acting on the original and rotated subunit.

4. Depending on whether or not we require the seed tile for assembly to be present at a particular point in the ruleset (thus making a particular tile special), the subunits comprising the minimal ruleset may be permuted around the available space in a genome. If a ruleset consists of n_t tiles, of which t are involved in bonding, this gives

$$s_4 = \begin{cases} \frac{(n_t-1)!}{(n_t-1-(t-1))!} & \text{for a specific seed tile,} \\ t\frac{(n_t-1)!}{(n_t-1-(t-1))!} & \text{for an arbitrary seed tile.} \end{cases} \quad (14)$$

5. Finally, other symmetry-preserving transformations may be made (e.g. `1230 3400` $\leftrightarrow$ `3120 4300`) depending on the phenotype structure: we will denote the number of transformations in this class by s_5.

The overall neutral space size is then:

$$\mathrm{NN}(\mathcal{P}|b, t, n_0, n_p, n_t, n_b) = s_1 s_2 s_3 s_4 s_5 r(l|n_0, n_p). \quad (15)$$

For the 2×1 domino in $\mathcal{S}_{2,8}$ ($n_0 = 2; n_c = 8$), we have the minimal ruleset `1000 2000`, so we have $b = 1, n_p = 4, l = 6$. Considering the above symmetric transformations that can be applied to the ruleset, we straightforwardly obtain $s_1 = 2$, $s_2 = 3$, $s_3 = 8$, $s_4 = 1$, $s_5 = 1$. Computing $r(6|2,4) = 13\,532$, we find that the overall neutral space size is 649 536.

For the 4×4 16-omino in $\mathcal{S}_{2,8}$, we have the minimal ruleset `1230 3400`. Properties of this ruleset immediately yield $b = 2, n_p = 2, l = 3$. Again, consideration of the symmetry transformations above give $s_1 = 4$, $s_2 = 6$, $s_3 = 16$, $s_4 = 1$, $s_5 = 2$. Computing $r(3|2,2) = 46$, we find that the overall neutral space size is 35 328.

Both of these neutral space sizes are in exact agreement with those found from exhaustively sampling the $\mathcal{S}_{2,8}$ space in Ref. [27]. These examples of exact calculations of the neutral space size associated with given phenotypes illustrate the quantitative link our theory describes between algorithmic complexity and neutral space. The minimal genome for the domino structure necessitates fewer interactions than for the 16-omino ($b = 1$ and $b = 2$ respectively), leading to more degrees of freedom associated with unused bond types ($n_p = 4$ and $n_p = 2$ respectively). Moreover, the lower number of sites in the domino genome occupied by necessary bonding information allows more freedom associated with remaining genetic loci ($l = 6$ and $l = 3$ respectively). What this combinatorial analysis shows is that, roughly, for a linear decrease in the minimal description length, we obtain an exponential increase in the number of ways that the phenotype can be encoded within the genomes. Thus these results help illustrate how complexity and neutral space are related to one another.

This model analysis sheds some light on how less complex phenotypes occupy more genetic space in other systems. For example, a certain number of amino acid residues are required in a protein sequence to create a binding interface supporting its quaternary structure role. Other residues, not involved in interfaces, are less constrained. If the protein requires more interfaces, more residues are constrained, leading to less redundancy and a smaller neutral space – qualitatively analogous to requiring more specified edges in the polyomino genomes above.

Finally, we note that other combinatorial approaches have successfully predicted the neutral set sizes for RNA, as well as the difference between the rank plots of systems like RNA, which do not have redundant parts of their genomes, and systems such as the polyominoes, which do [15, 28, 29]. One potential critique of the polyomino system is that the strong phenotype bias as well as the scaling of the NSS with complexity is caused by the redundant parts of the genome which are an artefact of the fixed length encoding we use. What these more general arguments show is that the same basic phenotype bias occurs both for systems with redundant parts of the genome, and for systems which do not have this feature. See also Fig. S7 for an example of the when the genome length is changed for polyominoes.

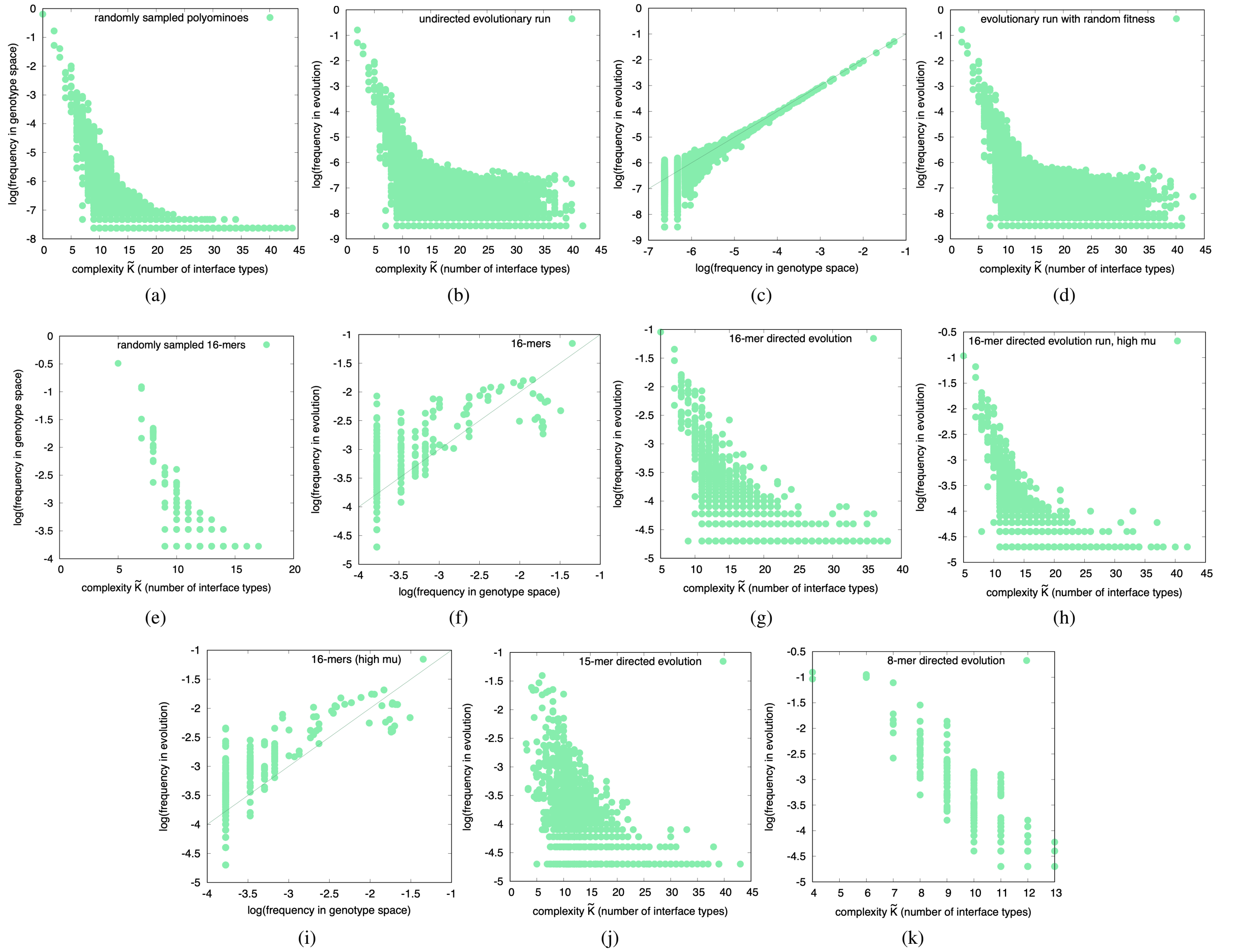

FIG. S6. **Polyomino frequency versus complexity plots for random sampling of genomes and for different evolutionary protocols.**
(a) Frequency (or equivalently occurrence probability $P(p) = NSS(p)/N_G$) with which (any size) polyomino structures appear upon random sampling of 10^8 genotypes from $\mathcal{S}_{16,64}$ decreases with complexity ($\tilde{K}(p)$ = number interface types), as expected from AIT coding theorem arguments [12]. Note that if a polyomino genome does not generate a fixed size polyomino structure deterministically, it is discarded.
(b) Frequency (probability) for that a particular polyomino structure appears during undirected evolutionary simulations (population $N = 100$ with a mutation rate $\mu = 0.1$, run for 5000 generations) versus complexity $\tilde{K}(p)$ looks similar to the sampled data in (a). Here the fitness of any deterministically assembly polyomino structure was set to 1, and the fitness of non-polyomino structures (either unbounded, or structures that were not deterministic) was set to zero.
(c) Frequencies from random sampling of genotypes (data from (a)) are directly compared to frequencies that a phenotype arises from the undirected evolution simulations of (b). The good agreement between random sampling and the evolutionary run demonstrates that a local evolutionary population encounters new variation with a probability on average close to the global $P(p)$ of phenotype p.
(d) Frequency (probability) that particular polyomino structure appears during versus complexity for an evolutionary run with random fitnesses protocol, where a random fitness value was applied to each polyomino shape, and reset for each evolutionary run.
(e) The (normalised) frequency/probability $P(p)$ with which 16-mer polyominoes appear upon random sampling of 10^8 genotypes from $\mathcal{S}_{16,64}$ decreases with complexity $\tilde{K}(p)$, as expected from AIT coding theorem arguments [12]. Note that these 16-mers are roughly one in 10^4 of all polyominoes found in (a).
(f) The probability that a 16-mer fixes in a directed evolution simulations with a fitness function with a maximum at size $s^* = 16$ for $N = 100$, mutation rate $\mu = 0.1$, and run for 5000 generations, correlates well (within statistical uncertainties) with the frequency of the same 16-mer structures, obtained by random sampling of genotypes.
(g) Frequency (probability) for the directed evolutionary run from (f), now plotted against complexity, shows the same global bias towards low complexity shapes as for direct sampling.
(h) Frequency (probability) for the directed evolutionary run for 16-mers, with $N = 100$ and a higher mutation rate $\mu = 1$ is very similar to the lower mutation rate results in (f).
(i) Comparing sampling of 16-mers to evolutionary simulations as in (f), but now for the high μ evolutionary run from (h).
(j) Directed evolutionary run for 15-mers for $N = 100$ and mutation rate $\mu = 0.1$ run for 5000 generations.
(k) Directed evolutionary run for 8-mers for $N = 100$ and mutation rate $\mu = 0.1$ run for 5000 generations.
Note that for all the plots above, statistical errors are largest for the lowest frequency structures.

C. Outcomes of evolutionary simulations with other mutation rates or fitness functions

In this section, we check that our main results for evolutionary dynamics from Fig. 1 in the main text are robust to changes in evolutionary parameters. The basics of our evolutionary simulations were described in the methods sections. Fig. S6 shows the results of a evolutionary simulations performed using a range of different evolutionary parameters and fitness functions.

In Figs. S6 (a)-(d) we study runs where any (bound and deterministic) phenotype (shape) is possible, i.e. the unit fitness protocol from Sec 1B. Thus, no particular structure is most fit, and so no structure fixes permanently. These simulations study the rate at which variation appears in an evolutionary run. Fig. S6 (a) shows the probability $P(p)$ that a phenotype of any size appears versus complexity $\tilde{K}(p)$ for random sampling of $\mathcal{S}_{16,64}$ genomes (the same data can be seen in Fig. 2 of the main text). Next, in Fig. S6 (b) we show the probability-complexity relationship for evolutionary simulations where all phenotypes have the same fitness. And in Fig. S6(c), we directly compare the frequency with which phenotypes appear in the evolutionary simulation to the probability that they appear upon random sampling. As can be clearly seen, the overall probability versus complexity relationships is quite similar to that obtained by random sampling of genotypes. There are differences at lower probabilities/higher complexities, but these may in part be due to statistical sampling issues. Note that the y-axis is on a log-scale, so that these phenotypes are found rarely, and that the majority of the probability weight is in the regime where the data from (a) and (b) are very similar. As argued above, and also in refs. [6, 9], the mean probability that a phenotype p appears in a population during an evolutionary run, which is fundamentally measuring a quantity which is *local* (i.e. it depends on the particular small subset of genotypes in the population), is very well approximated by the *global* frequency $P(p)$ which is the average over all genotypes. When this phenomenology is at play, then simple analytic forms such as those shown in Section S2 work well. Finally, in Fig. S6 (d) we show the outcome of simulations where where for each run, each bound, deterministic polyomino structure is assigned a fitness value uniformly distributed on $[0, 1]$, i.e. the *random fitness* protocol from Methods. Again, as expected within the arrival of the frequent picture, the probability-complexity relationships are remarkably similar to that obtained by random sampling of genotypes, and are also very close to the outcomes of the unit fitness protocol.

In Figs. S6(e)-(i), we show results for simulations and sampling with a focus on polyominoes of size $s = 16$, using the *size fitness* protocol from methods, where the fitness function for a polyomino of size s is $1/(|s - 16| + 1)$, so that polyominoes of size 16 have unit fitness and other sizes have fitness decreasing with distance from 16. First, in Fig. S6(e) we show the probability $P(p)$ that a 16-mer appears upon random sampling of $\mathcal{S}_{16,64}$ genomes vs. complexity $\tilde{K}(p)$. As expected from the coding theorem bound Eq. (17) (Eq.(1) in the main text), we once again see behaviour consistent with an upper bound that decreases exponentially for linear increases in complexity. Next, in Fig. S6(g) we compare the frequency with which 16-mers fix in an evolutionary simulation, to the probability that they appear upon random sampling (this data can also be seen in the inset of Fig. 2 of the main text). Although there is less data, so that the fluctuations are larger, we observe the same linear relation as seen in Fig. S6(c). Fig. S6(g) directly shows the evolutionary data for the fixation probability of 16-mers, which closely resembles the sampled data from Fig. S6(e). In Fig. S6(h) we show data for simulations with a much higher mutation rate μ, and finally, in Fig. S6(i) we directly compare the frequency with which 16-mers appear in an evolutionary simulation, to the probability that they appear upon random sampling. Again, as expected from predictions from Eqs.(1) and (2), the data is very similar to that found with the lower mutation rate, in fact the frequency versus complexity is slightly closer to the sampling results. This closer agreement may be due to the high μ: if evolutionary search is more randomised, the global effect of NSS on evolutionary observation will become ever stronger. Conversely, at low μ, evolution is more locally constrained, and effects from the local structure of search space can perhaps more easily drive the relationship away from simple global scaling with $P(p)$. More work is needed to confirm this conjecture.

The final two plots, Figs. S6(j) and (k), show the frequency versus complexity relationship for evolutionary runs for fitness functions $f = 1/(|s - 15| + 1)$, which favours 15-mers, and $f = 1/(|s - 8| + 1)$, which favours 8-mers. Both experiments show qualitatively similar behaviour to the runs for 16-mers.

Overall these results corroborate our claims in the main text, which is that the *arrival of the frequent* framework suggests that a highly biased GP map strongly constrains what variation can arise in practice, and that this results in evolutionary outcomes that strongly favour low complexity phenotypes over high complexity phenotypes. While this is the broad overall picture, a closer look at Fig. S6 suggests many additional subtle effects which would be interesting to explore in the future.

D. Symmetry and modularity in evolved polyominoes

1. *Enumeration of polyomino symmetries*

The distribution of symmetries of polyominoes have been characterised up to size $n = 27$. These data are available from the On-Line Encyclopedia of Integer Sequences, http://oeis.org/ and are curated and tabulated in Table I.

n	(a) D_4	C_4	D_2	C_2	D_1	C_1	(b) D_4	C_4	D_2	C_2	D_1	C_1
1	1	0	0	0	0	0	1	0	0	0	0	0
2	0	0	1	0	0	0	1	0	1	0	0	0
3	0	0	1	0	1	0	1	0	2	0	1	0
4	1	0	1	1	1	1	2	0	3	1	2	1
5	1	0	1	1	4	5	3	0	4	2	6	6
6	0	0	2	5	8	20	3	0	6	7	14	26
7	0	0	4	4	16	84	3	0	10	11	30	110
8	1	1	5	18	28	316	4	1	15	29	58	426
9	2	0	4	19	64	1196	6	1	19	48	122	1622
10	0	0	9	73	112	4461	6	1	28	121	234	6083
11	0	0	12	73	238	16750	6	1	40	194	472	22833
12	3	3	18	278	420	62878	9	4	58	472	892	85711
13	2	2	20	283	890	237394	11	6	78	755	1782	323105
14	0	0	35	1076	1595	899265	11	6	113	1831	3377	1222370
15	0	0	41	1090	3334	3422111	11	6	154	2921	6711	4644481
16	5	12	74	4125	6013	13069026	16	18	228	7046	12724	17713507
17	4	7	73	4183	12547	50091095	20	25	301	11229	25271	67804602
18	0	0	137	15939	22824	192583152	20	25	438	27168	48095	260387754
19	0	0	148	16105	47468	742560511	20	25	586	43273	95563	1002948265
20	12	44	292	61628	86832	2870523142	32	69	878	104901	182395	3873471407
21	7	25	273	62170	180531	11122817672	39	94	1151	167071	362926	14996289079
22	0	0	539	239388	332010	43191285751	39	94	1690	406459	694936	58187574830
23	0	0	540	240907	689858	1.68046E+11	39	94	2230	647366	1384794	2.26234E+11
24	20	165	1161	932230	1273985	6.54997E+11	59	259	3391	1579596	2658779	8.81231E+11
25	11	90	1026	936447	2647385	2.55722E+12	70	349	4417	2516043	5306164	3.43845E+12
26	0	0	2128	3641945	4907236	9.99908E+12	70	349	6545	6157988	10213400	1.34375E+13
27	0	0	2006	3651618	10197786	3.9153E+13	70	349	8551	9809606	20411186	5.25905E+13

TABLE I. **Symmetries of polyominoes of different sizes.** (a) Symmetry groups for polyominoes of size n. (b) Symmetry groups for all polyominoes of size up to and including n. Data taken from the On-Line Encyclopaedia of Integer Sequences, http://oeis.org/ under the following references: (all polyominoes) A000105; (D_4) A142886; (C_4) A144553; (D_2) A056877 + A056878 (diagonal and axial symmetry); (C_2) A006747; (D_1) A006746 + A006748 (diagonal and axial symmetry); (C_1) A006479.

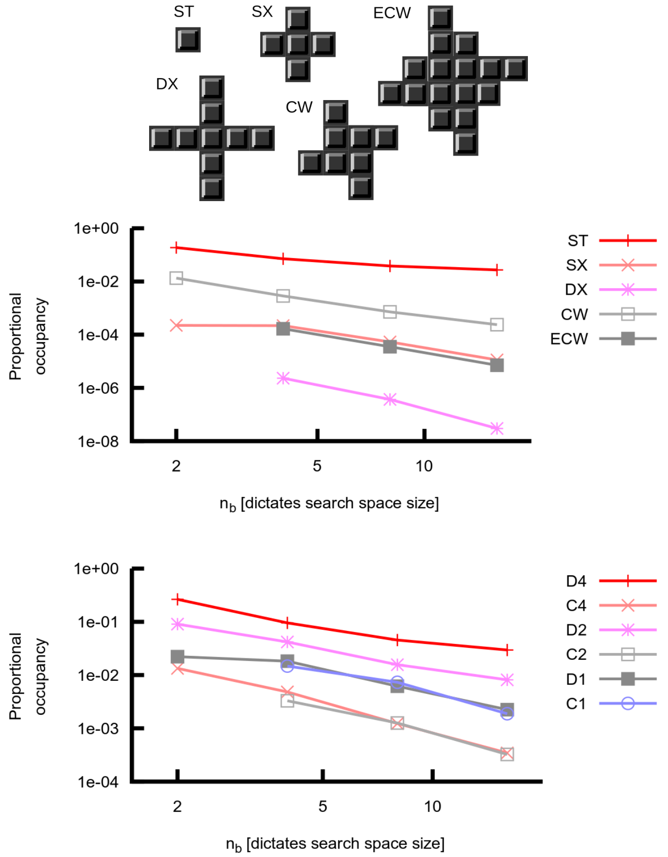

FIG. S7. **Proportion of genome space that encodes for a particular polyomino or symmetry group, versus the number of tiles $\mathbf{n_b}$ in spaces of the form $\mathcal{S}_{\mathbf{n_b},\mathbf{4n_b}}$ ranging from $\mathcal{S}_{\mathbf{2,8}}$ to $\mathcal{S}_{\mathbf{16,64}}$.** Top figure: some key tile shapes. ST, single tile; SX, single cross; DX, double cross; CW, 'catherine wheel'; ECW, extended catherine wheel. Middle figure, data denotes the frequency of individual tiles shown in the top figure. Bottom, the data are for all tiles of symmetries D_4, C_4, D_2, C_2, D_1 and C_1. Increasing the size of the space allows for more diversity in shapes, and in symmetries, as can also be seen in table 1. As n_b increases, the fraction of genomes leading to non-deterministic or unbounded structures also increases; this explains why the overall occupancies of the different polyomino structures decreases for larger spaces.

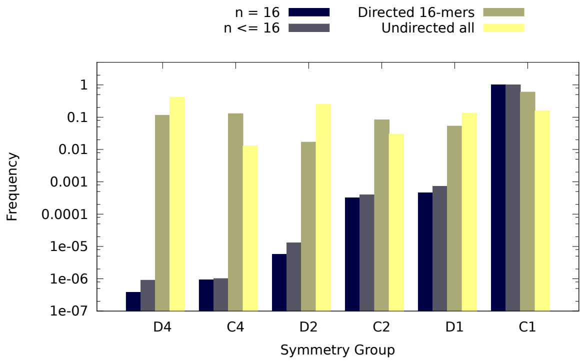

FIG. S8. **Comparison of symmetry groups for undirected evolutionary run and a run directed towards 16-mers** The two evolutionary simulations are for $N = 100, \mu = 0.1$ and run for 50,000 generations on $\mathcal{S}_{16,64}$. In one run, all finite self-assembling polyominoes have a fitness 1, and in the other directed evolutionary simulation a fitness maximum at size $s^* = 16$ is used. The frequencies for each symmetry group, found by the evolutionary simulations are compared to the overall frequencies from table I for $n = 16$ (black), and $n \leq 16$ (brown). The evolutionary simulations are very strongly biased towards high symmetry structures.

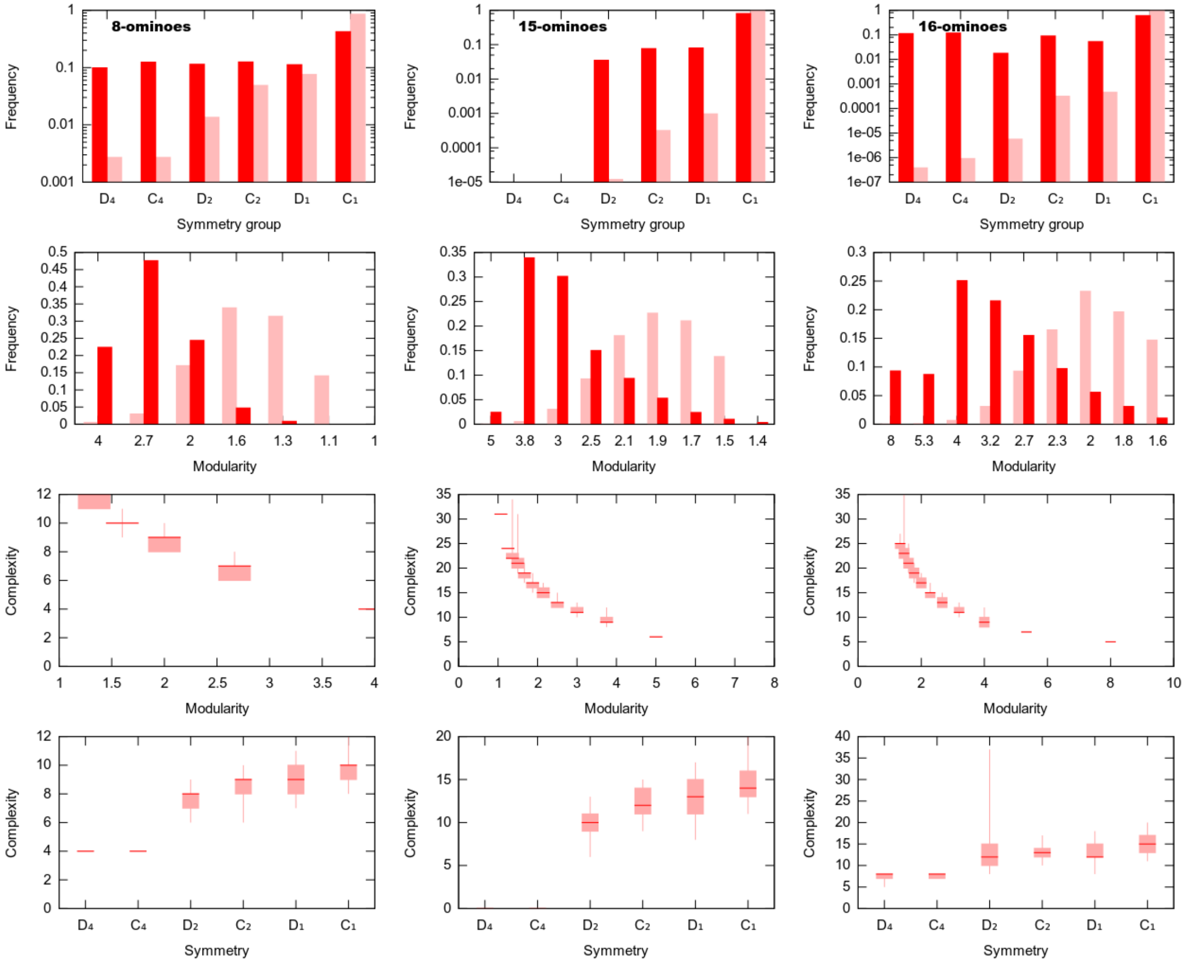

FIG. S9. **Symmetry, modularity and complexity in evolved polyomino structures.** Each column presents results of polyomino structures resulting from evolutionary simulation directed towards $s^* = 8, 15, 16$. (Row 1) Symmetry classes observed in evolved polyominoes (dark) compared to across all polyominoes of that size (light), showing a dramatic favouring of symmetric structures. (Row 2) Modularity index S/b observed in evolved structures, showing a favouring of higher-modularity structures up to a maximum index of $s^*/2$. (Row 3) Distribution of $\tilde{K}(p)$ complexity values (number of interface types) among polyominoes of a given modularity index shows a clear decrease of complexity with increasing modularity. Candlesticks show $0.05, 0.25, 0.5, 0.75, 0.95$ quantiles. (Row 4) Distribution of $\tilde{K}(p)$ complexity values among polyominoes of a given symmetry class shows a clear decrease of complexity with increasing symmetry. Candlesticks show $0.05, 0.25, 0.5, 0.75, 0.95$ quantiles.

2. *Scaling of symmetry group proportions with search space size*

How do our results hold across varying search space sizes? This is a consideration when interpreting our results in a biological context, as the genetic search spaces associated with organisms in nature are not of fixed size. For example, gene duplications and indels can vary the length of genomes. We explored the proportion of several differently sized search spaces occupied by genomes encoding specific polyomino structures, and the proportion occupied by genomes encoding structures that fall within various symmetry groups. Fig. S7 shows these proportions for search spaces ranging $\mathcal{S}_{2,8}$ to $\mathcal{S}_{16,64}$, covering many orders of magnitude in absolute search space size. As search space grows, we observe a general decrease in the proportion of search space encoding any given structure, which is unsurprising, as larger search spaces support more diverse structures. To first order, the proportion of high symmetry structures encoded by the genomes does not vary so much over this large variation in the size of the spaces.

3. *Symmetry and modularity of polyominoes from evolutionary simulation*

In Fig. S8 we display specific structures and symmetry classes observed in evolutionary simulation of polyomino structures, both undirected and directed towards $s^* = 16$. As can be easily seen, in both cases there is a very strong bias towards low complexity/high symmetry structures. The undirected runs have slightly more higher symmetry structures because a higher proportion of the smaller structures have higher symmetries (compare Table I (a) and (b)).

Next, in Figs. S9 (a)-(c) we compare the symmetries under evolutionary runs from above, to those from Table I(a) for 8-mers, 15-mers, and 16-mers. In each case we observe a strong bias towards symmetric structures.

In the following row, Figs. S9 (d)-(f) we compare a modularity index which is defined as the size s of the polyomino divided by the number of tile types n_b used in a minimal genome. This index measures how often units are repeated. For example, a modularity of 1 means every tile is different, whereas a modularity of $s/2$ means that just two tiles are used, on average $s/2$ times. Thus a higher modularity index means that the system is built out of a smaller number of modular units; one could say it is more modular (although biological modularity is a much more complex feature than just something that is repeated [30, 31]). As can be clearly seen in Figs. S9 (d)-(f), the evolutionary simulations are biased towards structures with a higher modularity index. We did not work out the modularity index of all structures, as that would require calculating the minimal rule sets of an enormous number of structures which is prohibitively expensive. Instead, we took all rule sets found in the simulation, and counted each structure once to obtain a distribution of modularity indices. If we had, instead, taken all structures for each size, then the bias towards high modularity would be (much) more pronounced.

In the next row, Figs. S9 (g)-(i), we compare our modularity index to complexity. As expected, the complexity increases with increasing modularity index. Note that the complexities are calculated for those structures that appear in the evolutionary simulation, which has a bias towards low complexity. In the final row, Figs. S9 (j)-(l), we compare symmetries to our complexity measure $\tilde{K}(p)$. As expected, the complexity increases with decreasing symmetry. Note that for these final two rows, the complexities are calculated for those structures that appear in the evolutionary simulation, which has a bias towards low complexity. If we were to compare the complexity and symmetry by uniformly sampling over all structures, then there would be more variation in the complexity.

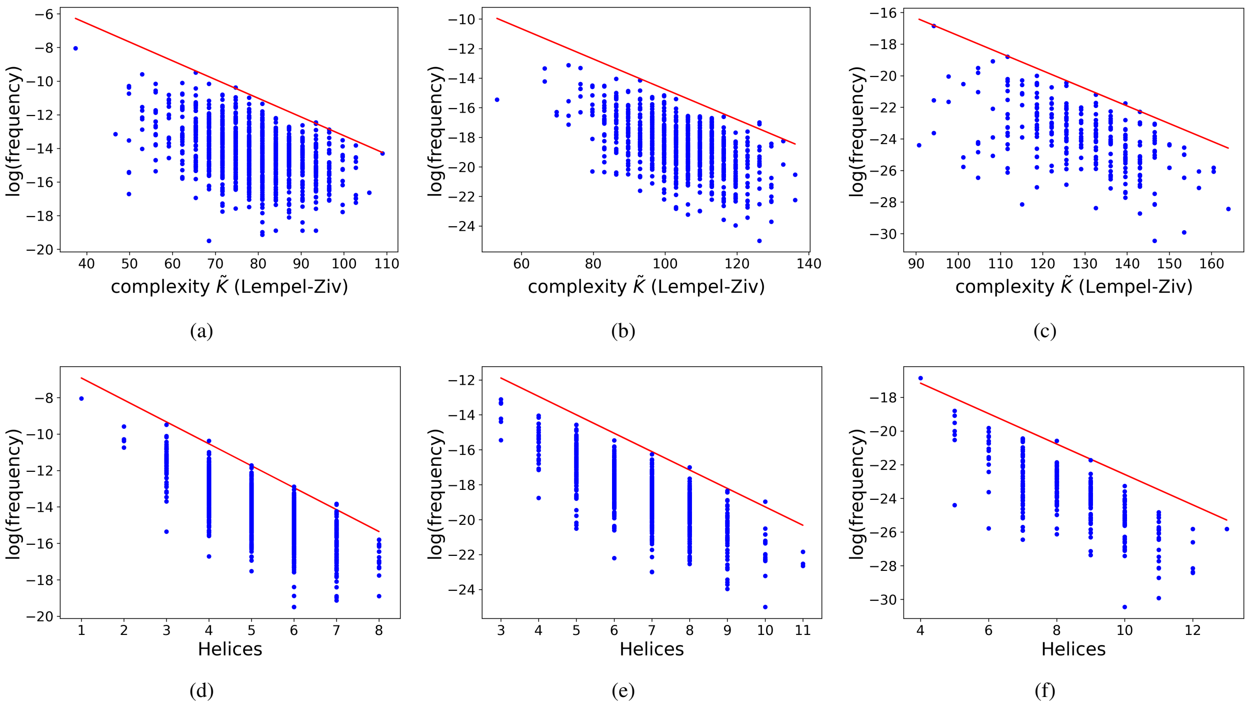

FIG. S10. Frequency (calculated using the NSSE neutral set size estimator [32]) versus complexity for natural RNA SS from the fRNAdb, for lengths $L = 75$, $L = 100$, and $L = 126$. There are 1419 sequences for $L = 75$, 932 for $L = 100$ and 318 for $L = 126$. The red line is an upper bound, Eq. 17 (Eq. (1) of the main text) where parameters a and b are fit. Complexity is measured in two ways: by C_{LZ} from Eq. (18) for (a) $L = 75$, $a = 0.37$ and $b = 7$; (b) $L = 100$, $a = 0.34$ and $b = 15$; and (c) $L = 126$, $a = 0.37$ and $b = 21$; and by the number of helices for (d) $L = 75$, $a = 4$ and $b = 19$; (e) $L = 100$, $a = 3.5$ and $b = 29$; (f) $L = 126$, $a = 3$ and $b = 45$. Both measures show the same overall scaling. The top probability structure for each value of the C_{LZ} complexity for $L = 100$ natural data in (b) is also shown in table III.

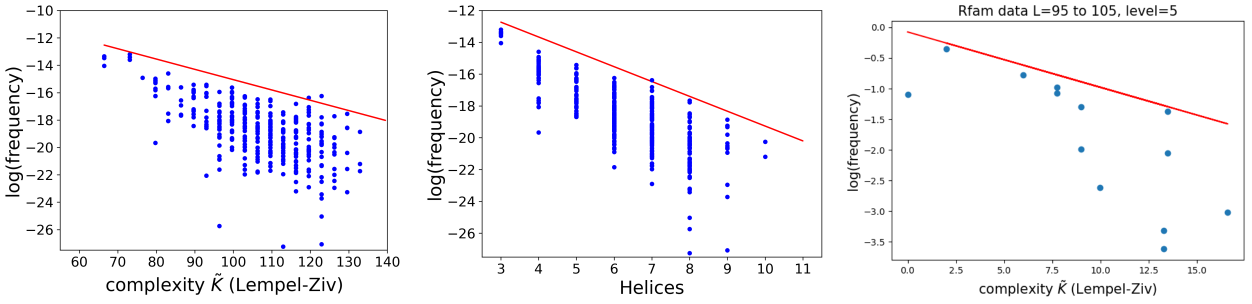

(a) (b) (c)

FIG. S11. Frequency versus complexity for natural RNA structures from the Rfam database [33, 34]. (a) $L = 100$ using the NSSE neutral set size estimator [32] to estimate frequencies versus complexity (measured by C_{LZ} from Eq. (18)) for natural RNA; The red line is an upper bound, Eq. 17 (Eq. (1) of the main text) consistent with the data. Parameters $a = 0.25$ and $b = 25$ are fit. (b) the same data as (a) but with complexity measured as number of helices ($a = 3.1$ and $b = 33$); (c) Level 5 coarse-grained structures for $L \approx 100$ (lengths $L = 95$ to 105 were binned together improve the reliability of database frequency estimates). There are 4124 sequences. As opposed to the other graphs in this section, the frequencies are directly taken from the Rfam database. Complexity is measured by C_{LZ}, but the structures come from consensus sequence alignment methods, not from folding calculations. The bound of Eq. (18) is for $a = 0.3$ and $b = 0.25$. The data is similar to that extracted from the fRNAdb in Fig. 3(b) of the main text. For all three Rfam data graphs, the same overall scaling of complexity with probability is observed as for the $L = 100$ data from the fRNAdb.

S4. SUPPLEMENTARY TEXT FOR RNA GP MAP

A. Probability-complexity for natural RNA from fRNAdb and Rfam with frequencies calculated with the NSSE

In Fig. S10 we plot frequency versus complexity $\tilde{K}(p)$ for natural RNA. The structures are computationally predicted from natural sequences taken from the fRNAdb database, and the probabilities are estimated using the NSSE from [32] to calculate the NSS for each sequence in the database. This method is the one that was used in ref. [17], and it is indirect. By contrast the frequencies/probabilities in the main text are directly sampled. Here we show the probability versus complexity measured in two ways. Firstly in (a)-(c) it is with the standard Lempel-Ziv measure from ref. [17], shown in Eq. (18). Secondly, for (d)-(f) we use a simpler measure, namely the number of helices or stacks, which has been proposed before as a complexity measure [35]. It also exhibits similar scaling to that expected from the upper bound of Eq. (17) (or equivalently, Eq. (1) in the main text). Note that the scaling for these longer lengths looks cleaner than in the main text for $L = 30$, where the shorter length means that finite size effects play a more important role.

As a quick check that database biases and structure prediction methods are not the main causal factor in our observations, in Fig. S11 we make probability-complexity plots for RNA with $L \approx 100$ data from the Rfam database [33, 34]. Fig. S11(a) and (b) show analogous plots to Fig. S10, with frequencies estimated via the NSSE [32], and complexity via C_{LZ} and via the number of helices.

Fig. S11(c) is a frequency-complexity plot where both the structures and the frequencies are derived via the same methods as in Fig. 3 of the main text. This method is different from the NSSE method used in Fig. S10: The frequencies are simply direct database frequencies of abstract shapes (level 5), and the secondary structures are obtained via consensus sequence alignment methods, which is fundamentally different from the energy minimization method used elsewhere in this work. The structures were taken from all available seed sequences of ncRNA families from the Rfam database (but some of the structures were ignored due to containing impossible/unusual motifs such as loops of zero length). We used lengths 95 to 105 and binned these together due to a paucity of data. Just as in the main text Fig. 3, abstract level 5 was used. It is evident that the same general relation of probability to complexity is observed as for the fRNAdb. This similarity is further support for the claim that the probability-complexity relationships are not due to database artefacts.

In Table II we show how our Lempel-Ziv complexity measure $C_{LZ}(x)$ from Eq. (1) in the main text (or Eq. (18) in these Supplementary Materials), correlates with the probability $P(p)$ that a particular RNA SS phenotype p obtains upon random sampling of sequences. The table also shows the dot-bracket notation of the SS, which helps illustrate what $C_{LZ}(x)$ is measuring. In Table III we show a similar plot, but now for natural $L = 100$ RNA from the fRNAdb, which are also shown in Fig. S10(b). Note that, as expected, the range of complexities in these natural RNAs is smaller than the fully sampled ones for $L = 55$ in Table II.

$C_{LZ}(p)$	$\log_{10} P(p)$	dot-bracket structure of SS
7	-0.13	....
31	-2.01	.....................................((((.....)))).....
34	-2.01	..((((((.....))))))....................................
37	-3.38	................(((((..(((((.......)))))..))))).........
41	-2.95	.....................(((..(((........)))..)))..........
44	-3.08	((((.(((((........)))))))))............................
44	-3.15	.............((((.(((.....)))))))......................
47	-2.81	..............................((((..(((.....)))..))))..
51	-2.99	..........................((((.(((........))).)))).....
54	-2.95	.............................((..((((.....))))..)).....
58	-3.48	..(((.((((((...............)))))).)))..................
61	-3.86	............((((..((((((.......)))))).)))).............
61	-5.06	(((.(((...))).)))............(((((....)))))............
64	-4.21	...........(((((.((....((((........))))....)).)))))....
64	-4.38	.(((((.((....((((......))))...)).))))).................
68	-4.29	...............(((.((..(((((.....)))))..)).))).........
71	-4.55	(((.((((...((((((.........))))))..)))).))).............
75	-5.32	.............(((((.(..((....(((......)))....))..).)))))
78	-5.84	(((.((.....(((...(((((....)))))..)))....)).))).........
78	-5.28	.............(((.((((..((.((((((....)))))).))..)))).)))
81	-6.01	((((..((((((....(((.(......).)))....))))))...))))......
85	-6.41	(((((...(((..(....)..)))...))))).((((.((....)).))))....
88	-6.54	...((.((((((..(..(((..((((....))))..)))..)..)))))).))..
88	-7.67	.(((((...(((((((.....)))).)))..)))))...((.(((....))).))
92	-7.94	((..((....))..)).((((((......))))))((.(((....))).))....
95	-7.46	((..(.....(((((((..(.((((....)))).)..))))))).....)..))

TABLE II. An example of how complexity $\tilde{K}(p) = C_{LZ}(p)$, probability $P(p)$ and the dot-bracket description of secondary structures for $L = 55$ RNA relate to one another. Structures were generated with the Vienna package [36] upon random sampling of sequences, and the probabilities were calculated with the NSSE [32]. For each complexity, the highest probability value SS was chosen. A clear decrease of probability with increasing complexity can be observed, which is consistent with Eq. (17).

B. RNA mutational robustness

The mutational robustness of a given structure p is defined as the fraction of random single point mutations of a random sequence underlying p, which map to p. The mutational robustness of RNA has been studied previously, and it is known that the robustness of a structure scales roughly linearly with the log probability of the structure [9, 15, 29]. Here we provide some further evidence for this scaling. Fig. S12(a) shows a roughly linear relation between mutational robustness and log frequency for $L = 18$. To make the plot, 10^6 samples were chosen, and all $3L$ single point mutations were enumerated, and each of these mutated sequences were folded to their associated structures. The linear relation is more clear for higher probability phenotypes. $L = 18$ was chosen as a small enough system that the linear relation becomes apparent, even on partial sampling. Next, Figs S12(b) and (c) show the same approximately linear relation, but now for $L = 30$ with 10^6 randomly sampled sequences and natural RNA data, respectively. There is more spread, in part because of the much larger number of sequences and structures, but to first order this scaling still holds.

It has been shownr [17] that for $L = 55$ ncRNA structures, the distribution of random and natural robustness values are virtually identical to one another, and quite different to the distribution obtained via directly sampling phenotypes (P-sampling). In Fig. S12(d), the directly calculated distribution of robustness values for $L = 30$ natural data and randomly sampled structures (G-sampled) are shown. Additionally, an estimate of the robustness distribution over all phenotypes (P-sampling) is given in black, which is obtained using the same methods described in ref. [17]. These calculations make use of accurate estimates for the log neutral set size distribution over all phenotypes, combined with a rough fit to the robustness-log probability plots for $L = 30$. Firstly, the natural and random distributions clearly differ significantly from the estimated P-sampled distribution over all structures. Secondly, the random and natural distributions are remarkably similar. Interestingly, in contrast to the case found in [17] for $L = 55$, where we found no statistical difference between natural data and randomly sampled data, for $L = 30$

$C_{LZ}(p)$	$\log_{10} P(p)$	dot-bracket structure of SS
53.15	-15.45	(((((((((((.((((((((..)))))))))))...))))))))..
66.44	-13.34	(((((((.((((((....)))))).........................(((((....))))).............................)))))))..
69.76	-16.30	(((((((.....................((((((.........((((..............))))))))))..((((((((....)))))))))))))))
73.08	-13.12	................((((((((..((((.......))))...(((((((........)))))))).......))))))))...................
76.40	-13.30	....(((((.........(((((((....)))))))..........................(((((((..............)))))))..)))))...
79.73	14.50	...................((((((((((((.......................(((.....)))......(((......)))...))))))).))))).
83.05	-14.51	((((((((.(....((((.....)))).).))))))))...............................(((((.......)))))..............
86.37	-14.05	..........((((((((.((...............((((((....))))))......((((((.....)))))).)).))))))))............
89.69	-14.86	........((((..(((.((((((((...(((((((.......................))))))).)))))))).)))....)))).............
93.01	-14.15	.............(((.(((((((....((((.........))))..........((((((.......))))))..))))))).))).............
96.34	15.27	(((((.....))))).((((((.....((((.((((((........)))))).)))).....))))))...((((....)))).................
99.66	-15.16	(((.((((....)))).))).....((((((...(((....))).)))))).................. ((((((..................))))))
102.98	-15.41	(((....(((((((........(((((((((....)))))))))..(((.(((.....))).)))...................)))))))....)))..
106.30	-16.55	(((((((...(((.(((((((.((.((((((..(((.((.......)))))..)))))).)).))))))).)))...)))))))................
109.62	-16.59	.(((.(((...((((((((.......((((.((((....)))).))))(((((....)))))..............)))))))).....))).)))...
112.95	-16.70	....(((((((..(((.........)))...)).)))))..(((((((...(((((.....)))))...)))))))........((((....))))....
116.27	-16.61	((.((((.((((((.((..(((..........((((....)))).....(((((....))))).............)))..)).)))))))))).))..
119.59	-17.69	(((..((.((((((.((((((....(((....)))).........((..(((((....)))))..))..........)))))).))))))))..)))..
122.91	-17.61	((....((..((((.((..(((.........((((((....))))))(((((....)))))................)))..)).)))).))....))..
126.23	-17.00	............((.(((((......((((....))))..((((((.((.(((.(..(((......)))..).))).))))))))....))))).))...
129.56	-18.43	((.(((...(((.((((..(.(((((.((((..(((....)))..)))).))))).)..))))..)))..))).)).....(((((......)))))...
132.88	-18.26	.(((.(..((((............((((((.((.((((..((((((.(((.........))).))))))..)))).))....))))))))))..).))).
136.20	-20.52	....((....(((((.((((((((.((.(..((.((((..........)))).))..).))))))..))))....(((....))).))))).....))..

TABLE III. An example of how complexity $\tilde{K}(p) = C_{LZ}(p)$, database frequency $P(p)$ and the dot-bracket description of secondary structures for naturally occurring $L = 100$ RNA relate to one another. The structures are from fRNAdb, and for each one, the probabilities were calculated with the NSSE [32]. For each complexity, the highest probability value SS was chosen. The full set of probabilities and complexities can be seen in Fig. S10(b). A clear decrease of probability with increasing complexity can be observed, as expected from Eq. (17) and as can also be seen in Fig. S10(b)

we observe that the natural data has slightly higher mean robustness values: 0.36 for the natural data vs. 0.32 for the random samples. While this difference in means is statistically significant (two sample t-test p-value$< 10^{-100}$), the absolute difference is relatively small (on the order of 10%). Nevertheless, this result suggests that there may have been a small amount of natural selection for higher robustness in these shorter strands. The P-sampled mean robustness is 0.18, considerably less than either the random or natural mean robustness values.

One possible alternative explanation for the overall correlation we find between frequency and neutral set size is that there exists a strong selection for robustness. While it is true that a selection for robustness would lead to deviations from P-sampling, it would be extremely surprising if this led to the overall distribution of robustness being so close to the randomly G-sampled one, as seen for $L = 55$ [17] and $L = 30$ structures here. Thus, we believe that this potential alternative hypothesis is highly unlikely to hold.

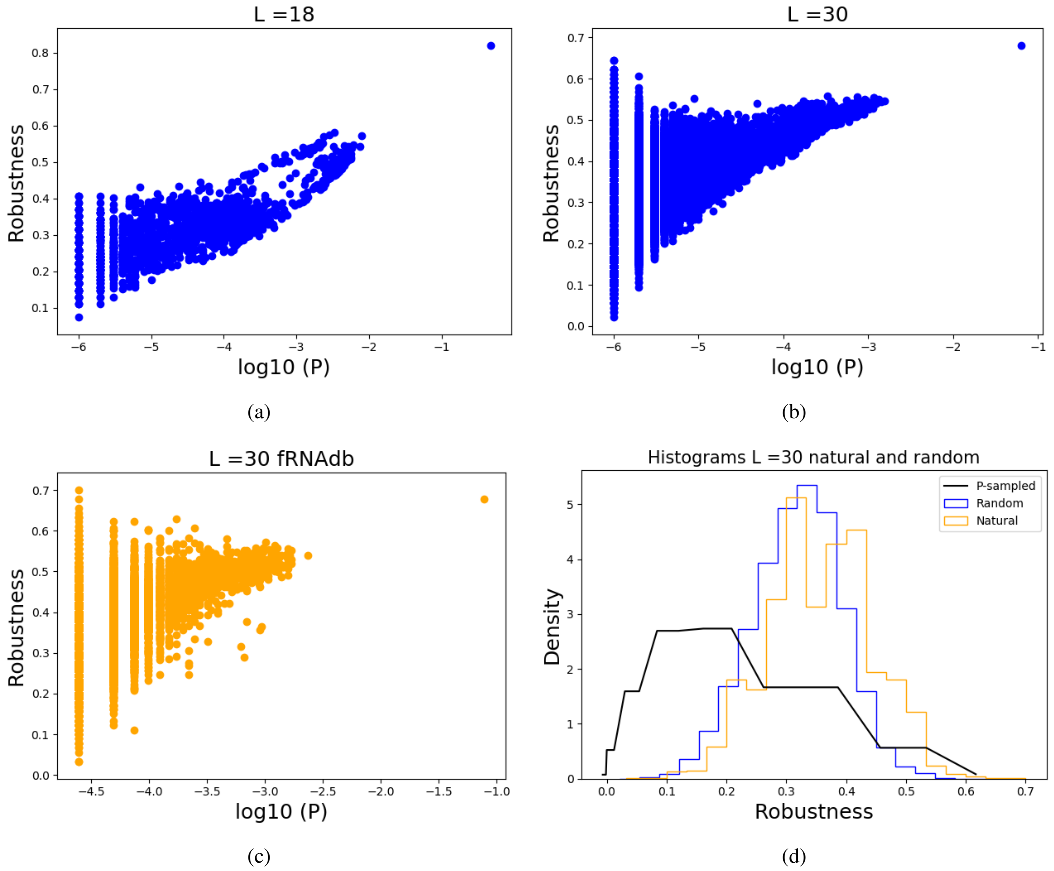

FIG. S12. RNA mutational robustness plots: (a) A roughly linear relation between robustness and log probability for randomly sampled RNA, $L = 18$; (b) A roughly linear relation between robustness and log probability for randomly sampled RNA, $L = 30$ and (c) natural fRNAdb $L = 30$ data; (d) Natural and random RNA $L = 30$ have very similar robustness distributions, and markedly different from the P-sampled (direct phenotype sampled) distribution. A very similar close correlation between randomly sampled structures and natural structures was shown for $L = 55$ [17], although here we see that there is also a small but statistically significant difference between natural data and G-smapled data (marked Random in the graph).

S5. SUPPLEMENTARY TEXT FOR ALGORITHMIC INFORMATION THEORY AND COMPLEXITY MEASURES

A. Algorithmic information theory, Kolmogorov complexity and the AIT coding theorem

The word *complexity* is used in many different ways across science (see e.g. ref. [37] for an overview), and this wide variation in meaning often causes confusion. In biological contexts measures of complexity related to Shannon information theory, which is fundamentally a theory about distributions, have been widely applied [35, 38–40]. An alternative approach is to use algorithmic information theory (AIT), which is a different concept since it is a theory about individual objects, although there are deep links between Shannon information and AIT [41]. Here we focus on measures related to Kolmogorov complexity, a key concept from AIT, which formally captures the intuition that strings with short descriptions have lower complexity than strings that only allow long descriptions. Consider, for example the two 100 digit long binary strings below:

```
0101010101010101010101010101010101010101010101010101010101010101010101010101010101010101010101010101
01000001100100110001111010001111101001101000010101011110001000100000100011011100111010001101101001101
```

The first string intuitively has low Kolmogorov complexity because it can be described by a small program such as "print "01" 50 times", while the second string has, as far as we know, no shorter program than to simply print the full string. More formally, Kolmogorov complexity $K(x)$ is defined as the length of the shortest program that generates a string x on a suitably chosen (optimal) universal Turing machine (UTM – a model computer capable of simulating any other). Since a UTM can mimic any other Turing machine, there is an *invariance theorem* [41, 42] which states that if $K_U(x)$ and $K_V(x)$ are the Kolmogorov complexities defined with respect to UTMs U and V respectively, then $|K_U(x) - K_V(x)| \leq M_{U,V}$, where $M_{U,V}$ is a constant independent of p. The actual UTM used is then often informally dropped, and so we speak simply of $K(x)$. Nevertheless, it is

important to remember that Kolmogorov complexity is always defined with respect to a particular UTM (see ref. [41] for more in-depth discussion).

An important link between Kolmogorov complexity and probability was derived by Levin [43], building upon earlier work on algorithmic probability by Solomonoff [44] who in fact provided the first formulation of what is now called Kolmogorov or Kolmogorov-Chaitin complexity. Very briefly (for more technical details, see e.g. ref. [41]) the AIT coding theorem states that the probability $P(x)$ that a randomly selected (binary) input program fed into a (optimal prefix) UTM generates output x is bounded by

$$2^{-K(x)} \leq P(x) \leq 2^{-K(x)+\mathcal{O}(1)} \tag{16}$$

where $K(x)$ is the Kolmogorov complexity of output x. The (unknown) $\mathcal{O}(1)$ term is independent of x, and hence in an asymptotic regime of large complexities, can be ignored. Informally, what Eq. (16) tells us is that upon randomly choosing programs, a UTM is exponentially more likely to produce outputs with low $K(x)$ than outputs with high $K(x)$.

Despite its profound implications, the coding theorem has not been widely applied in science and engineering (see however, [45]). One of the reasons is that many practical systems are not UTMs, and the coding theorem (like much of AIT) depends on the computational power of UTMs. The second reason is that the Kolmogorov complexity $K(x)$ is formally uncomputable due to the famous halting problem of UTMs, first pointed out by Turing [46]. In a recent paper [12] that was inspired by the AIT coding theorem, a practically useable upper bound for the $P(x)$ that output x obtains upon uniform sampling of inputs was derived for input-output maps $f : I \to O$ that are *simple*, that is $K(f)$ is independent of, or grows very slowly with, the size of the output space. It takes the form

$$P(x) \leq 2^{-a\tilde{K}(x)-b}\,. \tag{17}$$

where $\tilde{K}(x)$ is a suitable approximation to the Kolmogorov complexity of output x, and a and b are constants that are independent of x, and which can often be determined from some basic information about the map. Note that, in contrast to the full coding theorem (16), Eq. (17) only provides an upper bound. Nevertheless, a statistical lower bound can be derived [12, 47] showing that most of the probability weight in $P(x)$ will be close to the bound (17). Interestingly, outputs that are far from the bound can be shown to have inputs (in this case genomes or bonding patterns) that themselves are unusually simple [47]. Since the number of such simple inputs is a tiny fraction of all inputs, this is another way of showing that outputs far from the bound will have, on average, low probability.

B. A Lempel-Ziv based compression approximation for $\tilde{\mathbf{K}}(\mathbf{p})$

How to best choose a computable complexity measure that approximates the true Kolmogorov complexity is not an easy question to answer, see for example refs. [41, 48–51]) and further discussion in the Supplementary Information of ref. [12]. There is a deep connection between Kolmogorov complexity and compression. For the RNA and GRN, we use a compression method by Lempel and Ziv [52] in which a (binary) string x is compressed by looking for patterns (words). The number of words $N_w(x)$ forms the basis for this complexity measure, and it has been a popular choice for approximating Kolmogorov complexity in the literature. In particular, it is thought to work better than many rival methods for shorter strings [53, 54]. In ref. [12] a Lempel-Ziv based complexity measure was defined as follows

$$C_{LZ}(x) = \begin{cases} \log_2(n), & x = 0^n \text{ or } 1^n \\ \log_2(n)[N_w(x_1...x_n) + N_w(x_n...x_1)]/2, & \text{otherwise,} \end{cases} \tag{18}$$

where $N_w(x_1...x_n)$ is the dictionary produced by the Lempel-Ziv algorithm, and $(x_1...x_n)$ denotes the values of the string x of length n. The simplest strings 0^n and 1^n are separated out because $N_w(x_1....x_n)$ assigns complexity $K = 1$ to the string 0 or 1, but complexity 2 to 0^n or 1^n for $n \geq 2$, whereas the true Kolmogorov complexity of such a trivial string actually scales as $\log_2(n)$, as one only needs to encode n. In ref. [12, 25, 47] this complexity measure was empirically shown to work well for a wide diversity of maps, ranging from sets of coupled differential equations, to the RNA SS GP map, to a finite-state transducer, to neural network models for deep learning. This success gives us confidence to use it here for the RNA and the GRN. We note that in the Supplementary Information of ref. [12] we compare a number of other compression based measures for RNA, finding a similar scaling of $P(p)$ versus $\tilde{K}(p)$ for all of them (note that we use $\tilde{K}(p)$ when we are specifically referring to the complexity of some phenotype p, whereas we use $\tilde{K}(x)$ when referring to some general output x). Similarly, in ref. [25] we compare a wide range of different complexity measures for a fully connected deep learning model of Boolean functions, finding again that these different measures all correlate with one another, giving us confidence that our results are not that sensitive to the exact method used to estimate or bound the Kolmogorov complexity.

C. Alternative complexity measures for the polyominoes

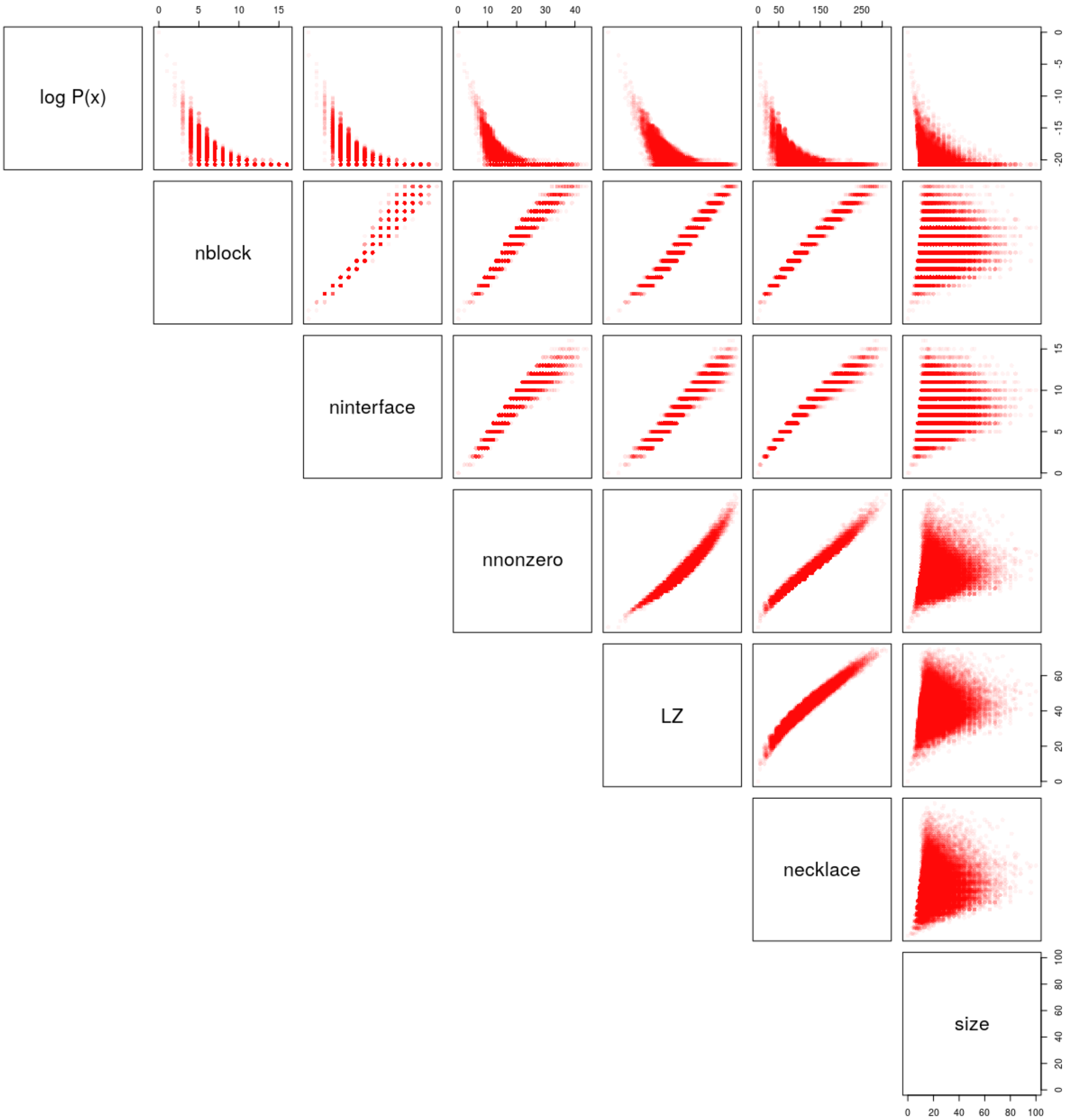

FIG. S13. A comparison of different complexity measures for polyominoes for polyomino phenotypes that arise from random sampling of the genotypes in $\mathcal{S}_{16,64}$; The measures are:
– nblock = the number n of tiles (blocks);
– ninterface = the number of labels used in the required interfaces;
– nnonzero = the number of non-zero elements of the minimal genome (this directly measures the amount of novel mutations needed, which is equivalent to the number of interface types. This measure of complexity is used in the main text));
– LZ = our Lempel-Ziv based measure $C_{LZ}(p)$ from Eq. (18);
– necklace = a combinatorial complexity measure from ref. [55] based on necklaces, which can be defined as equivalence classes of strings under rotation.
The complexity measures all generate qualitatively similar $P(p)$ versus $\tilde{K}(p)$ plots, and also correlate well with one another. They all appear to capture some basic properties of the descriptional complexity of the polyominoes. The final row is not a complexity measure, but rather the size of the polyominoes, which does not correlate that well with the complexity measures, as expected, because some large structures can be assembled from relatively simple instruction sets. Nevertheless, smaller sizes are more likely to appear than larger sizes, as can be seen in the top right panel. This is because they typically have shorter instruction sets, and so are more likely to appear because fewer evolutionary innovations are needed to make them.

For the polyominoes, we also explored several other complexity measures $\tilde{K}(p)$ that are all related to the Kolmogorov complexity. These include the number of tiles (blocks), the number of interfaces, the number of non-zero elements of the minimal genome (this directly measures the amount of novel mutations needed, as in the Main Text), our Lempel-Ziv measure from Eq. (18), and a measure from ref. [55] based on necklaces, which can be defined as equivalence classes of strings under rotation. These measures are all compared in Fig. S13 for the polyomino data from Fig. 2 of the main text, that is random sampling of the genotypes. We note that the measures all correlate well with one another supporting our assumption that the exact details of the complexity measure is not critical. They all exhibit the expected exponential scaling between probability and complexity that is predicted by the AIT inspired coding theorem of Eq. (1) in the main text. By contrast, the size of the polyominoes does not correlate as well with any of the measures, as expected.

While the more sophisticated Lempel-Ziv complexity measure (18) is straightforward to apply to the minimal assembly kit genomes for polyominoes, it is not clear how to use this measure for the protein complexes. In order to compare the polyominoes with the protein complexes, we therefore use the simplified measure that counts the number of interface types. Intuitively, this correlates with the amount of new information that mutations need to supply in order to create a self-assembling polyomino or protein structure, and indeed in Fig. S13 we see that it correlates well with the full Lempel-Ziv complexity measure.

We note that in an important paper [56] Soloveichik and Winfree show that the minimum number of distinct tiles to make a shape scales with the Kolmogorov complexity of that shape. There is a link to our complexity measure because the number of interface types correlates with the number of distinct tile types. Indeed, for the polyominoes, our complexity measure based on the number of tiles correlates closely with the one based on the number of interfaces (see Fig. S13). Further work is needed to flesh out these connections. For the protein complexes, the number of interfaces is a more natural measure than the number of "tiles".

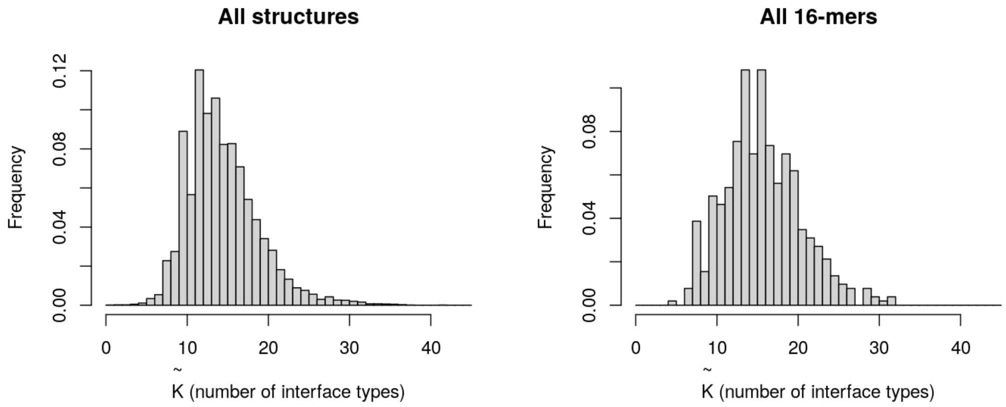

FIG. S14. **Complexity histograms for sampled polyominoes.** The data is the same as in Fig 2 of the main text, obtained by randomly sampling 10^8 genotypes for the $\mathcal{S}_{16,64}$, but now it is plotted with all polyominoes of a given complexity (number of interface types) counted in each bin. In this way we create histograms of the frequency of occurrence of each complexity bin (which is equivalent to $P(K)$). (Left) All structures. (Right) only 16-mer structures. While the vast majority of polyominoes phenotypes have high complexity, the bias towards simple polyominoes is strong enough that low to intermediate complexities are the most likely to appear. A qualitatively similar trend can be see in Fig S9 of this supplementary materials, and in Fig 1 F of the main text, where instead of complexity, we have the symmetry groups plotted on the x-axis.

S6. PROBABILITY VERSUS COMPLEXITY HISTOGRAMS

In this paper we have have mainly considered probability versus the complexity relationships for individual phenotypes. In this subsection we consider instead the probability $P(K)$ of obtaining phenotypes of a given complexity K, for the case of G-sampling of sequences, or for the case of evolutionary dynamics of a population.

On the one had, the upper bound given in Eq. (17) predicts that high probability phenotypes are typically simple (they have relatively low descriptional complexity $\tilde{K}(p)$). On the other hand, there are good reasons to expect that many if not most phenotypes in a GP map system have relatively high complexity. This expectation arises from basic counting arguments. For example, most binary strings have a Kolmogorov complexity close to their length in bits. There are 2^n different strings of length n in $\{0,1\}^n$, and $2^n - 1$ shorter strings in the set $\{0,1\}^{\leq(n-1)}$. Thus at least one string cannot be fully compressed. Moreover, 2^{n-1} of these strings are are just one bit shorter, so at best, 50% of the strings in $\{0,1\}^n$ can be compressed by at most one bit,

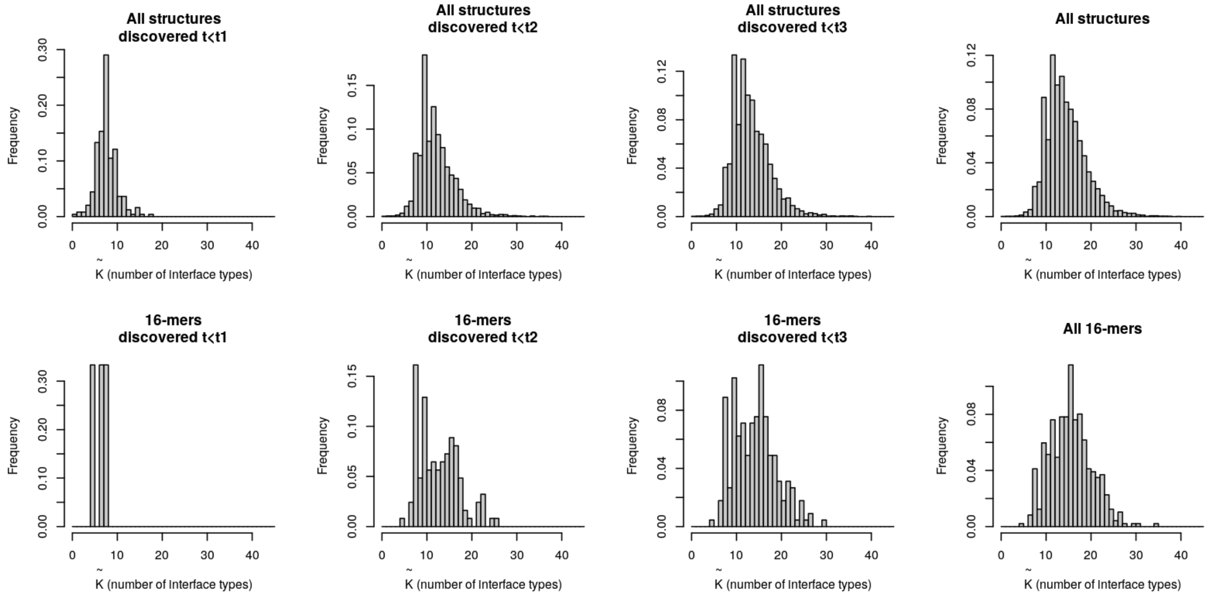

FIG. S15. **Complexity histograms as a function of generations for polyomino simulations with undirected evolutionary dynamics.** Simulations are for $\mathcal{S}_{16,64}$ polyominoes, with population size $N = 100$ and mutation rate $\mu = 0.1$, as in Fig. S6. Here the fitness of any deterministically assembling polyomino structure was set to 1, and the fitness of non-polyomino structures (either unbounded, or structures that were not deterministic) was set to zero. We count the number of times a shape of complexity $\tilde{K}(x)$ appears, and so create histograms of the occurrence of each complexity bin. By row: (i) all individual polyominoes (ii) all 16-mers From left to right: distributions plotted for structures with a minimum discovery time under different thresholds: $t_1 = 1000, t_2 = 5000, t_3 = 10000$ generations or the full 20000 generations. It takes longer for more complex phenotypes to start to appear. Note how similar the final results are to the directly sampled results of fig. S14.

75% by at most two bits, etc.... This means that most strings cannot be compressed by much, and so most are close to maximal $K(x)$.

The arguments for strings above predicts that the number of strings of complexity K scales as 2^K. As a null model, the $2^{-K(x)}$ scaling from the coding theorem for the probability of obtaining a single output of complexity $K(x)$ should more or less counterbalance the 2^K growth of the number of outputs of complexity K so that as a null model, we expect a roughly uniform probability $P(K)$ of obtaining any particular complexity K.

For GP maps these scaling arguments are more complicated because not all theoretically conceivable phenotypes may be possible in practice. For example, there are dot-bracket SS patterns that may not be the lowest free-energy state for any RNA sequence etc.... Nevertheless, the exponential nature of the counting argument above means that we still expect that most dot-bracket phenotypes will have high complexity. This can be observed explicitly in systems such as $L = 20$ RNA where all phenotypes can be enumerated [6]. Similarly, the vast majority of polyomino shapes of a given size have the lowest symmetry. For example, 99.9% of all polyomino 16-mers have C_1 symmetry, which is the lowest one. Since symmetry correlates with complexity, this suggests that the vast majority of polyominoes shapes are relatively complex. A second reason why a G-sampled GP map may not exactly obtain the null-model expectation of a flat $P(K)$ distribution is that Eq. (17) is only an upper bound. Some individual probabilities are well below the bound. For these reasons, the exact probability distribution of the complexities may differ from the null model. Nevertheless, this null model suggests that the distribution should be significantly different from what one would get if instead one sampled uniformly over phenotypes (P-sampling), where the probability should grow rapidly (most likely exponentially) with increasing complexity.

In Fig. S14, we plot, for polyominoes, the normalised histograms of the probability versus complexity, taken from the G-sampled polyomino data from Fig. 2 of the main text. Figures are shown for all structures, and for just for 16-mers. While, as shown in Fig. 2 of the main text, the lowest complexity polyominoes have the highest individual frequencies upon random sampling, there are only few of them, and so the maximum in $P(K)$ is for an intermediate value. For higher values the drop in individual frequencies is strong enough to counteract the rapid growth in the number of possible polyominoes, and so the probability of obtaining high complexity polyominoes by random sampling of sequences is low.

We can also study how complexity changes with time (numbers of generations) in evolutionary simulations. In Fig. S15 we show such data for undirected evolutionary simulations (as in Fig. S6b). That is, we simply count every possible polyomino that occurs during a simulation. Similar results are found for the temporal behaviour of the complexity distributions under other evolutionary protocols from Fig. S6 and so these are not plotted here. Since individual complex phenotypes have a lower

probability to be discovered, the average and maximum complexity grows with time. From the arrival of the frequent formalism (see e.g. eqns. (3) and (4)) we expect that to first order, the maximum complexity found grows as $K_{max} \sim \log(t)$. Longer simulations are needed to confirm this exact scaling, and this will be the subject of future investigations.

In Fig. S16 we show the probability versus complexity histograms for the $L = 30$ RNA system data from Fig. 3A of the main text. Randomly sampled sequences generates distributions that are similar to those found in the fRNA database [57]. Both show a fairly even distribution over the full range of complexities observed. For the $L = 100$ data from Fig 3 of the main text we did not have enough data to produce useful complexity histograms.

Finally, as shown in Fig. S17, we also find qualitatively similar behaviour for the budding-yeast GRN coupled ODE system from [58]. Again a range of complexities are found, but the rapid decay of the probability of individual phenotypes is strong enough to counteract the growth in the number of phenotypes with increasing complexity.

In conclusion, the results from these three systems suggest that the following patterns may more generally true for GP maps: 1) Even though the number of phenotypes grows rapidly with complexity, simplicity bias means that the probability of the system generating an individual complex phenotype of any kind remains relatively low. 2) Even though individual phenotypes at the lowest complexities typically have the highest individual frequencies, because there are very few low-complexity phenotypes, the most likely complexity will typically be at an intermediate value. 3) We expect that the maximum complexity of phenotypes discovered by an evolving population grows slowly with increasing numbers of generations in evolutionary runs. Further work is needed to confirm the nature and robustness of these patterns across other GP maps.

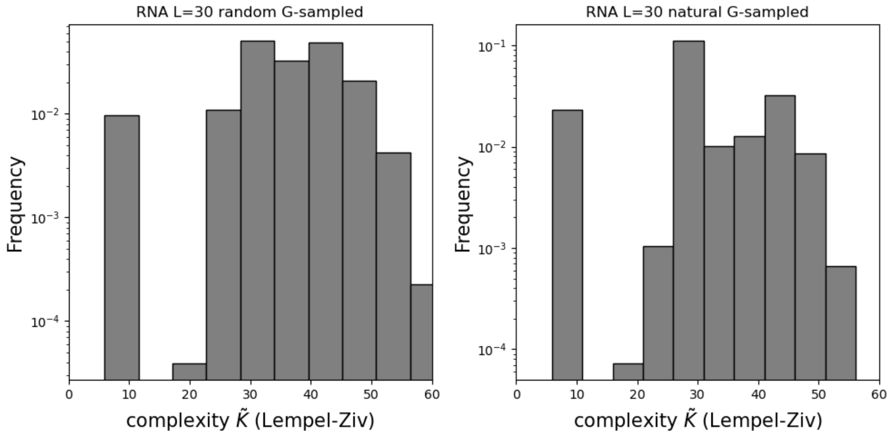

FIG. S16. **Complexity histograms for $\mathbf{L = 30}$ RNA.** The data is the same as in Fig 3A of the main text, but now it is plotted with all RNA dot-bracket SS of a given complexity counted in each bin. In this way we create histograms of the occurrence of each complexity bin. (Left) Data from 10^5 randomly sampled sequences. (Right) Data from 40,554 functional $L = 30$ sequences in the RNA database [57] which map to 17,603 unique dot-bracket SS. The majority of RNA SS have high complexity. Nevertheless, because simple RNA SS are much more likely to occur upon random sampling of sequences, their enhanced probability counteracts the growth in the number of possible SS with increasing complexity.

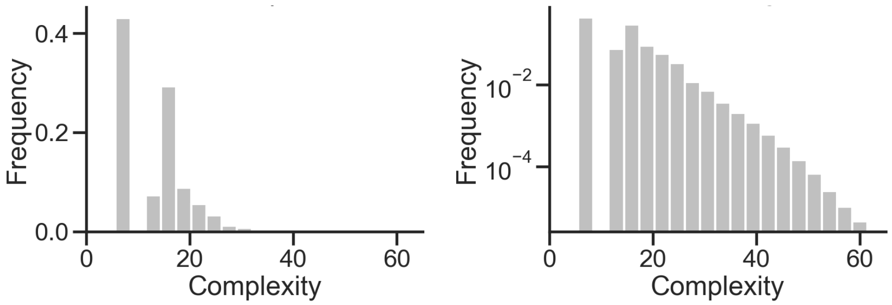

FIG. S17. **Complexity histograms for budding yeast cell-cycle.** The data is the same as in Fig 4 of the main text, but now it is plotted with all GRN outputs of a given complexity counted in each bin. In this way we create histograms of the frequency for each complexity bin. (Left) Frequency v.s. complexity. (Right) Log of the frequency v.s. complexity helps accentuate the low frequency/high complexity outputs. Again, while the number of possible complex outputs is much larger than the number of low complexity outputs, the bias towards simplicity means that high complexity outputs remain rare upon random sampling of genotypes.

S7. SUPPLEMENTARY TEXT FOR BIAS IN OTHER GP MAPS

1. *Protein tertiary structure*

Proteins fold into well-defined three-dimensional structures. Although the folding problem from sequence to structure is highly complex, there are simplified models that can be used to investigate the overall structure of the GP map. A particularly popular model in this regard has been the highly simplified HP lattice model, where folds are represented as self-avoiding walks on a lattice, and the full sequence is reduced to binary alphabet (H stands for hydrophobic and P for polar amino acids) [59]. Despite its heavily coarse-grained nature, this model has produced important biological insights [60]. In particular, a study by Li *et al* [61] showed that for both 2D and 3D models the $NSS(p)$ distribution was highly biased. For example, in the 3 by 3 by 3 cubic lattice model (with 51704 structures), the number of sequences per structure ranged from $\sim$4000 progressively down to only $\sim$1. Interestingly, it was found that the structures with the largest NSS (the most designable ones) showed tertiary symmetries and geometrical regularities, similar to the regular and symmetrical forms of natural protein folds, see also [62]. This observation fits within the framework of our observation of the symmetry and simplicity present in large NSS polyominoes, proteins complexes, and RNA structures. From a different perspective, England and Shakhnovich [63] and later Coluzza *et al.* [64] have used analytical methods to predict bias in natural protein tertiary structure maps. We note that in this literature, the word "designability" is used to describe how many genotypes (sequences) map to a phenotype (typically a fold). So the simplicity-bias framework predicts that more designable structures will have lower descriptional complexities.

2. *Target of rapamycin signalling circuit*

Raman and Wagner [65] studied a systems biology model of the target-of-rapamycin (TOR) signalling circuit for budding yeast (*Saccharomyces cerevisiae*). TOR is a highly conserved protein kinase which controls growth in yeast, fly and mamalian cells. The aim of their study was to see how the topology of the circuit interactions affects the signalling circuit's behaviour. The circuit's phenotypes were determined on the basis of the concentration-time trajectories of eight key proteins complexes. Specifically, the model generates a continuous concentration-time profile for each protein, and these were discretised by recording the concentrations at a fixed number of time points. Finally, these trajectories were clustered into similar signalling behaviours using a well known clustering algorithm used for large data sets. The algorithm clustered the (coarse grained) signalling behaviours of key signalling molecules into 286 qualitatively different behaviours, which were then taken as 286 different phenotypes. They then found that this GP map shows very strong bias. For example, the largest $NSS(p)$ contains 21633 genotypes, or 31% of the total $\sim$70000 genotypes and only 26 phenotypes account for nearly all (82%) of the genotypes. While their method does not obviously allow a complexity measure, it is interesting that it exhibits such strong bias. It would be interesting to attempt to measure the complexity of the phenotypes for this system and others like it.

3. *Boolean threshold models of GRNs*

Boolean models of GRNs, where individual genes are simplified to nodes having only an on or off state, and where the temporal dynamics is controlled by a set of Boolean rules for interactions with other nodes (genes), were first introduced by Kauffman [66]. In spite of the great simplification, they have been successfully applied to a wide range of biological phenomena, including segment polarity in *Drosophila* [67], flower development [68], signal transduction in human fibroblasts [69], plant cell signalling [70], and mammalian cortical development [71]. In addition, these models have been used to study more general properties of GRNs, including mutational robustness [72–75] and versions of the concept of evolvability [73–76].

Nevertheless, the state space of Boolean models grows very rapidly with the number of nodes and so it is often hard to achieve good sampling over genotypes. A popular simplification of these models is to use a thresholding rule to determine whether the inputs to a particular node (gene) result in the gene being turned off or on. Such Boolean threshold models (BTNs), which mathematically closely resemble neural networks, have also been successfully used to model GRNs. Examples include GRNs for the mammalian cell cycle [77], the regulation of lymphocyte differentiation [78], and signal transduction in human fibroblasts [69]. Applications of BTNs to the modelling the yeast cell cycle [79–81], have successfully led to predictions of knockout mutant phenotypes [81, 82]. The models have also been used to provide explanations for the designability (redundancy) and robustness of the wild-type phenotype [83–85]. Given their success, it is interesting to think about BTNs as GP maps [10], where the nodes and connections describe the genotypes, and the dynamic behaviour of the model is treated as its phenotype.

In fact, inspired by earlier discoveries of phenotypic bias in HP model lattice proteins [61], Nochomvitz and Li [86] presented evidence for strong bias in BTNs. It has recently been shown that these BTNs also exhibit clear simplicity bias [10], as well as log-linear robustness-frequency behaviour for the mutational robustness, and other properties that are very similar to those observed for other GP maps [14, 16]. Given that we also observed simplicity bias in an ODE system in the main text (where the connectivity is fixed, but the connetions vary in strength), the fact that it is also seen in this quite different and more simplified

type of modelling (where the connectivity is varied) suggest that the question of simplicity bias in GRNs may be ripe for further exploration.

4. Model of neuron development

Psujek and Beer performed an investigation of phenotypic bias in a computational model of neuronal development [87]. Due to the computational intractability of large neuron networks, the system they developed consists of only three neuron cells, and each pair of neurons i and j may have a connection arrow (neuronal connection) going from i to j, from j to i, or none at all. The phenotype is the connectivity pattern of these neurons, and hence there are $2^6 = 64$ possible phenotypes. The authors observed phenotypic bias on sampling genotypes; interestingly they noted that simpler connection patterns appeared more frequently, which is similar to what we observe in our work with polyominoes, proteins and RNA structures. This behaviour is not surprising in light of more recent papers showing that the perceptron [88] and several architectures for deep neural networks [25] show clear simplicity bias.

[1] Sebastian E Ahnert, Joseph A Marsh, Helena Hernández, Carol V Robinson, and Sarah A Teichmann. Principles of assembly reveal a periodic table of protein complexes. *Science*, 350(6266):2245, 2015.
[2] Emmanuel D Levy, Jose B Pereira-Leal, Cyrus Chothia, and Sarah A Teichmann. 3d complex: a structural classification of protein complexes. *PLoS computational biology*, 2(11):e155, 2006.
[3] Joseph A Marsh and Sarah A Teichmann. Structure, dynamics, assembly, and evolution of protein complexes. *Annual review of biochemistry*, 84:551–575, 2015.
[4] E. D. Levy, E. B. Erba, C. V. Robinson, and S. A. Teichmann. Assembly reflects evolution of protein complexes. *Nature*, 453:1262, 2008.
[5] Gabriel Villar, Alex W Wilber, Alex J Williamson, Parvinder Thiara, Jonathan PK Doye, Ard A Louis, Mara N Jochum, Anna CF Lewis, and Emmanuel D Levy. Self-assembly and evolution of homomeric protein complexes. *Physical review letters*, 102(11):118106, 2009.
[6] Steffen Schaper and Ard A Louis. The arrival of the frequent: how bias in genotype-phenotype maps can steer populations to local optima. *PloS one*, 9(2):e86635, 2014.
[7] Arlin Stoltzfus. Understanding bias in the introduction of variation as an evolutionary cause. *arXiv preprint arXiv:1805.06067*, 2018.
[8] Arlin Stoltzfus. *Mutation, Randomness, and Evolution*. Oxford University Press, 2021.
[9] Sam F Greenbury, Steffen Schaper, Sebastian E Ahnert, and Ard A Louis. Genetic correlations greatly increase mutational robustness and can both reduce and enhance evolvability. *PLoS computational biology*, 12(3):e1004773, 2016.
[10] Chico Q Camargo and Ard A Louis. Boolean threshold networks as models of genotype-phenotype maps. In *Complex Networks XI*, pages 143–155. Springer, 2020.
[11] Pablo Catalán, Susanna Manrubia, and José A Cuesta. Populations of genetic circuits are unable to find the fittest solution in a multilevel genotype–phenotype map. *Journal of the Royal Society Interface*, 17(167):20190843, 2020.
[12] Kamaludin Dingle, Chico Q Camargo, and Ard A Louis. Input–output maps are strongly biased towards simple outputs. *Nature communications*, 9(1):761, 2018.
[13] Jacobo Aguirre, Javier M Buldú, Michael Stich, and Susanna C Manrubia. Topological structure of the space of phenotypes: the case of rna neutral networks. *PloS one*, 6(10):e26324, 2011.
[14] Sebastian Edmund Ahnert. Structural properties of genotype–phenotype maps. *Journal of The Royal Society Interface*, 14(132):20170275, 2017.
[15] Juan Antonio García-Martín, Pablo Catalán, Susanna Manrubia, and José A Cuesta. Statistical theory of phenotype abundance distributions: A test through exact enumeration of genotype spaces. *EPL (Europhysics Letters)*, 123(2):28001, 2018.
[16] Susanna Manrubia, José A Cuesta, Jacobo Aguirre, Sebastian E Ahnert, Lee Altenberg, Alejandro V Cano, Pablo Catalán, Ramon Diaz-Uriarte, Santiago F Elena, Juan Antonio García-Martín, et al. From genotypes to organisms: State-of-the-art and perspectives of a cornerstone in evolutionary dynamics. *Physics of Life Reviews*, 37, 2021.
[17] Kamaludin Dingle, Steffen Schaper, and Ard A Louis. The structure of the genotype–phenotype map strongly constrains the evolution of non-coding RNA. *Interface focus*, 5(6):20150053, 2015.
[18] Kamaludin Dingle, Fatme Ghaddar, Petr Šulc, and Ard A Louis. Phenotype Bias Determines How Natural RNA Structures Occupy the Morphospace of All Possible Shapes. *Molecular Biology and Evolution*, (msab280), 09 2021.
[19] C. O. Wilke, J. Wang, C. Ofria, R. E. Lenski, and C. Adami. Digital organisms: survival of the flattest. *Nature*, 412:331–333, 2001.
[20] David M McCandlish and Arlin Stoltzfus. Modeling evolution using the probability of fixation: history and implications. *The Quarterly review of biology*, 89(3):225–252, 2014.
[21] L.Y. Yampolsky and A. Stoltzfus. Bias in the introduction of variation as an orienting factor in evolution. *Evolution & Development*, 3(2):73–83, 2001.
[22] Yann LeCun, Yoshua Bengio, and Geoffrey Hinton. Deep learning. *nature*, 521(7553):436–444, 2015.
[23] Shangshang Yang, Ye Tian, Cheng He, Xingyi Zhang, Kay Chen Tan, and Yaochu Jin. A gradient-guided evolutionary approach to training deep neural networks. *IEEE Transactions on Neural Networks and Learning Systems*, 2021.

[24] Felipe Petroski Such, Vashisht Madhavan, Edoardo Conti, Joel Lehman, Kenneth O Stanley, and Jeff Clune. Deep neuroevolution: Genetic algorithms are a competitive alternative for training deep neural networks for reinforcement learning. *arXiv preprint arXiv:1712.06567*, 2017.
[25] Guillermo Valle-Pérez, Chico Q Camargo, and Ard A Louis. Deep learning generalizes because the parameter-function map is biased towards simple functions. *arXiv preprint arXiv:1805.08522*, 2018.
[26] Chris Mingard, Guillermo Valle-Pérez, Joar Skalse, and Ard A Louis. Is sgd a bayesian sampler? well, almost. *Journal of Machine Learning Research*, 22(79):1–64, 2021.
[27] Iain G Johnston, Sebastian E Ahnert, Jonathan P K Doye, and Ard A Louis. Evolutionary dynamics in a simple model of self-assembly. *Physical Review E*, 83(6):066105, 2011.
[28] SF Greenbury and Sebastian Edmund Ahnert. The organization of biological sequences into constrained and unconstrained parts determines fundamental properties of genotype–phenotype maps. *Journal of The Royal Society Interface*, 12(113):20150724, 2015.
[29] Marcel Weiß and Sebastian E Ahnert. Phenotypes can be robust and evolvable if mutations have non-local effects on sequence constraints. *Journal of The Royal Society Interface*, 15(138):20170618, 2018.
[30] G.P. Wagner, M. Pavlicev, and J.M. Cheverud. The road to modularity. *Nature Reviews Genetics*, 8(12):921–931, 2007.
[31] Hod Lipson. Principles of modularity, regularity, and hierarchy for scalable systems. *Journal of Biological Physics and Chemistry*, 7(4):125, 2007.
[32] T. Jorg, O.C. Martin, and A. Wagner. Neutral network sizes of biological RNA molecules can be computed and are not atypically small. *BMC bioinformatics*, 9(1):464, 2008.
[33] Ioanna Kalvari, Joanna Argasinska, Natalia Quinones-Olvera, Eric P Nawrocki, Elena Rivas, Sean R Eddy, Alex Bateman, Robert D Finn, and Anton I Petrov. Rfam 13.0: shifting to a genome-centric resource for non-coding rna families. *Nucleic acids research*, 46(D1):D335–D342, 2018.
[34] Ioanna Kalvari, Eric P Nawrocki, Joanna Argasinska, Natalia Quinones-Olvera, Robert D Finn, Alex Bateman, and Anton I Petrov. Non-coding rna analysis using the rfam database. *Current protocols in bioinformatics*, 62(1):e51, 2018.
[35] J.M. Carothers, S.C. Oestreich, J.H. Davis, and J.W. Szostak. Informational complexity and functional activity of RNA structures. *Journal of the American Chemical Society*, 126(16):5130–5137, 2004.
[36] I.L. Hofacker, W. Fontana, P.F. Stadler, L.S. Bonhoeffer, M. Tacker, and P. Schuster. Fast folding and comparison of RNA secondary structures. *Monatshefte für Chemie/Chemical Monthly*, 125(2):167–188, 1994.
[37] Seth Lloyd. Measures of complexity: a nonexhaustive list. *IEEE Control Systems Magazine*, 21(4):7–8, 2001.
[38] C. Adami, C. Ofria, and T.C. Collier. Evolution of biological complexity. *Proceedings of the National Academy of Sciences of the United States of America*, 97(9):4463, 2000.
[39] C. Adami. The use of information theory in evolutionary biology. *Annals of the New York Academy of Sciences*, 1256:49–65, 2012.
[40] Andreas Wagner. Information theory, evolutionary innovations and evolvability. *Philosophical Transactions of the Royal Society B: Biological Sciences*, 372(1735):20160416, 2017.
[41] M. Li and P.M.B. Vitanyi. *An introduction to Kolmogorov complexity and its applications*. Springer-Verlag New York Inc, 2008.
[42] TM Cover and J.A. Thomas. *Elements of information theory*. John Wiley and Sons, 2006.
[43] L.A. Levin. Laws of information conservation (nongrowth) and aspects of the foundation of probability theory. *Problemy Peredachi Informatsii*, 10(3):30–35, 1974.
[44] Ray J Solomonoff. A formal theory of inductive inference. part i. *Information and control*, 7(1):1–22, 1964.
[45] Fernando Soler-Toscano, Hector Zenil, Jean-Paul Delahaye, and Nicolas Gauvrit. Calculating Kolmogorov complexity from the output frequency distributions of small Turing machines. *PloS one*, 9(5):e96223, 2014.
[46] Alan Mathison Turing. On computable numbers, with an application to the entscheidungsproblem. *J. of Math*, 58(345-363):5, 1936.
[47] Kamaludin Dingle, Guillermo Valle Pérez, and Ard A Louis. Generic predictions of output probability based on complexities of inputs and outputs. *Scientific reports*, 10(1):1–9, 2020.
[48] C. Adami and N. J. Cerf. Physical complexity of symbolic sequences. *Physica D*, 137(1):62–69, 2000.
[49] J.P. Delahaye and H. Zenil. Numerical evaluation of algorithmic complexity for short strings: A glance into the innermost structure of algorithmic randomness. *Appl. Math. Comput.*, 219:63–77, 2012.
[50] Hector Zenil, Fernando Soler-Toscano, Kamaludin Dingle, and Ard A Louis. Correlation of automorphism group size and topological properties with program-size complexity evaluations of graphs and complex networks. *Physica A: Statistical Mechanics and its Applications*, 404:341–358, 2014.
[51] Hector Zenil, Liliana Badillo, Santiago Hernández-Orozco, and Francisco Hernández-Quiroz. Coding-theorem like behaviour and emergence of the universal distribution from resource-bounded algorithmic probability. *International Journal of Parallel, Emergent and Distributed Systems*, 34(2):161–180, 2019.
[52] A. Lempel and J. Ziv. On the complexity of finite sequences. *Information Theory, IEEE Transactions on*, 22(1):75–81, 1976.
[53] José M Amigó, Janusz Szczepański, Elek Wajnryb, and Maria V Sanchez-Vives. Estimating the entropy rate of spike trains via lempel-ziv complexity. *Neural Computation*, 16(4):717–736, 2004.
[54] Annick Lesne, Jean-Luc Blanc, and Laurent Pezard. Entropy estimation of very short symbolic sequences. *Physical Review E*, 79(4):046208, 2009.
[55] S. E. Ahnert, I. G. Johnston, T. M. A. Fink, J. P. K. Doye, and A. A. Louis. Self-assembly, modularity, and physical complexity. *Physical Review E*, 82(2):026117, 2010.
[56] David Soloveichik and Erik Winfree. Complexity of self-assembled shapes. *SIAM Journal on Computing*, 36(6):1544–1569, 2007.
[57] Taishin Kin, Kouichirou Yamada, Goro Terai, Hiroaki Okida, Yasuhiko Yoshinari, Yukiteru Ono, Aya Kojima, Yuki Kimura, Takashi Komori, and Kiyoshi Asai. frnadb: a platform for mining/annotating functional rna candidates from non-coding rna sequences. *Nucleic Acids Research*, 35(suppl 1):D145–D148, 2007.

[58] K.C. Chen, L. Calzone, A. Csikasz-Nagy, F.R. Cross, B. Novak, and J.J. Tyson. Integrative analysis of cell cycle control in budding yeast. *Molecular Biology of the Cell*, 15(8):3841, 2004.

[59] Ken A Dill. Theory for the folding and stability of globular proteins. *Biochemistry*, 24(6):1501–1509, 1985.

[60] A. Wagner. *The Origins of Evolutionary Innovations: A Theory of Transformative Change in Living Systems*. Oxford University Press, 2011.

[61] H. Li, R. Helling, C. Tang, and N. Wingreen. Emergence of preferred structures in a simple model of protein folding. *Science*, 273(5275):666, 1996.

[62] Tairan Wang, Jonathan Miller, Ned S Wingreen, Chao Tang, and Ken A Dill. Symmetry and designability for lattice protein models. *The Journal of Chemical Physics*, 113(18):8329–8336, 2000.

[63] J.L. England and E.I. Shakhnovich. Structural determinant of protein designability. *Physical review letters*, 90(21):218101, 2003.

[64] Ivan Coluzza, James T MacDonald, Michael I Sadowski, William R Taylor, and Richard A Goldstein. Analytic markovian rates for generalized protein structure evolution. *PloS one*, 7(5):e34228, 2012.

[65] K. Raman and A. Wagner. Evolvability and robustness in a complex signalling circuit. *Mol. BioSyst.*, 7:1081–1092, 2011.

[66] Stuart Kauffman. Homeostasis and differentiation in random genetic control networks. *Nature*, 224(5215):177–178, 1969.

[67] Réka Albert and Hans G Othmer. The topology of the regulatory interactions predicts the expression pattern of the segment polarity genes in Drosophila melanogaster. *Journal of theoretical biology*, 223(1):1–18, 2003.

[68] Carlos Espinosa-Soto, Pablo Padilla-Longoria, and Elena R Alvarez-Buylla. A gene regulatory network model for cell-fate determination during Arabidopsis thaliana flower development that is robust and recovers experimental gene expression profiles. *The Plant Cell*, 16(11):2923–2939, 2004.

[69] Tomáš Helikar, John Konvalina, Jack Heidel, and Jim A Rogers. Emergent decision-making in biological signal transduction networks. *Proceedings of the National Academy of Sciences*, 105(6):1913–1918, 2008.

[70] Song Li, Sarah M Assmann, and Réka Albert. Predicting essential components of signal transduction networks: a dynamic model of guard cell abscisic acid signaling. *PLoS biology*, 4(10):e312, 2006.

[71] Clare E Giacomantonio and Geoffrey J Goodhill. A Boolean model of the gene regulatory network underlying mammalian cortical area development. *PLoS computational biology*, 6(9):e1000936, 2010.

[72] Ricardo BR Azevedo, Rolf Lohaus, Suraj Srinivasan, Kristen K Dang, and Christina L Burch. Sexual reproduction selects for robustness and negative epistasis in artificial gene networks. *Nature*, 440(7080):87, 2006.

[73] Stefano Ciliberti, Olivier C Martin, and Andreas Wagner. Innovation and robustness in complex regulatory gene networks. *Proceedings of the National Academy of Sciences*, 104(34):13591–13596, 2007.

[74] Stefano Ciliberti, Olivier C Martin, and Andreas Wagner. Robustness can evolve gradually in complex regulatory gene networks with varying topology. *PLoS computational biology*, 3(2):e15, 2007.

[75] Christopher F Steiner. Environmental noise, genetic diversity and the evolution of evolvability and robustness in model gene networks. *PLOS ONE*, 7(12):e52204, 2012.

[76] Aviv Bergman and Mark L Siegal. Evolutionary capacitance as a general feature of complex gene networks. *Nature*, 424(6948):549, 2003.

[77] Adrien Fauré, Aurélien Naldi, Claudine Chaouiya, and Denis Thieffry. Dynamical analysis of a generic Boolean model for the control of the mammalian cell cycle. *Bioinformatics*, 22(14):e124–e131, 2006.

[78] Elisabeth Remy, Paul Ruet, Luis Mendoza, Denis Thieffry, and Claudine Chaouiya. From logical regulatory graphs to standard petri nets: dynamical roles and functionality of feedback circuits. In *Transactions on Computational Systems Biology VII*, pages 56–72. Springer, 2006.

[79] Fangting Li, Tao Long, Ying Lu, Qi Ouyang, and Chao Tang. The yeast cell-cycle network is robustly designed. *Proceedings of the National Academy of Sciences of the United States of America*, 101(14):4781–4786, 2004.

[80] Maria I Davidich and Stefan Bornholdt. Boolean network model predicts cell cycle sequence of fission yeast. *PLOS ONE*, 3(2):e1672, 2008.

[81] Maria I Davidich and Stefan Bornholdt. Boolean network model predicts knockout mutant phenotypes of fission yeast. *PLOS ONE*, 8(9):e71786, 2013.

[82] Gunnar Boldhaus, Nils Bertschinger, Johannes Rauh, Eckehard Olbrich, and Konstantin Klemm. Robustness of Boolean dynamics under knockouts. *Physical Review E*, 82(2):021916, 2010.

[83] Gunnar Boldhaus and Konstantin Klemm. Regulatory networks and connected components of the neutral space. *The European Physical Journal B-Condensed Matter and Complex Systems*, 77(2):233–237, 2010.

[84] Hao Chen, Guanyu Wang, Rahul Simha, Chenghang Du, and Chen Zeng. Boolean models of biological processes explain cascade-like behavior. *Scientific reports*, 7, 2016.

[85] Neşe Aral and Alkan Kabakçıoğlu. Coherent organization in gene regulation: a study on six networks. *Physical biology*, 13(2):026006, 2016.

[86] Y.D. Nochomovitz and H. Li. Highly designable phenotypes and mutational buffers emerge from a systematic mapping between network topology and dynamic output. *Proceedings of the National Academy of Sciences of the United States of America*, 103(11):4180, 2006.

[87] S. Psujek and R.D. Beer. Developmental bias in evolution: evolutionary accessibility of phenotypes in a model evo-devo system. *Evolution & Development*, 10(3):375–390, 2008.

[88] Chris Mingard, Joar Skalse, Guillermo Valle-Pérez, David Martínez-Rubio, Vladimir Mikulik, and Ard A Louis. Neural networks are a priori biased towards boolean functions with low entropy. *arXiv preprint arXiv:1909.11522*, 2019.
